# A Head-Mounted Camera System Integrates Detailed Behavioral Monitoring with Multichannel Electrophysiology in Freely Moving Mice

**DOI:** 10.1016/j.neuron.2018.09.020

**Published:** 2018-10-10

**Authors:** Arne F. Meyer, Jasper Poort, John O’Keefe, Maneesh Sahani, Jennifer F. Linden

**Affiliations:** 1 Gatsby Computational Neuroscience Unit, University College London (UCL), London W1T 4JG, UK; 2 Sainsbury Wellcome Centre for Neural Circuits and Behaviour, UCL, London W1T 4JG, UK; 3 Department of Cell and Developmental Biology, UCL, London WC1E 6BT, UK; 4 Ear Institute, UCL, London WC1X 8EE, UK; 5 Department of Neuroscience, Physiology and Pharmacology, UCL, London WC1E 6BT, UK

**Keywords:** eye movement, head movement, pupil, whisker, pinna, oculomotor, vestibular, natural behavior, visual cortex

## Abstract

Breakthroughs in understanding the neural basis of natural behavior require neural recording and intervention to be paired with high-fidelity multimodal behavioral monitoring. An extensive genetic toolkit for neural circuit dissection, and well-developed neural recording technology, make the mouse a powerful model organism for systems neuroscience. However, most methods for high-bandwidth acquisition of behavioral data in mice rely upon fixed-position cameras and other off-animal devices, complicating the monitoring of animals freely engaged in natural behaviors. Here, we report the development of a lightweight head-mounted camera system combined with head-movement sensors to simultaneously monitor eye position, pupil dilation, whisking, and pinna movements along with head motion in unrestrained, freely behaving mice. The power of the combined technology is demonstrated by observations linking eye position to head orientation; whisking to non-tactile stimulation; and, in electrophysiological experiments, visual cortical activity to volitional head movements.

## Highlights

• Eyes, whiskers, head, and neural activity monitored in freely moving mice
• System generates stable video output and leaves mouse behavior largely unchanged
• Close link between eye and head movements at both slow and fast timescales
• Active head movements in the dark strongly modulate primary visual cortex activity

## Teaser

Meyer et al. describe a new head-mounted camera system that enables detailed behavioral monitoring along with neural recording in freely moving mice. The system reveals close coupling between eye and head movements, and head-movement-related activity in primary visual cortex.

## Introduction

A fundamental goal of neuroscience is to understand how neural circuits integrate a wide range of inputs to produce flexible and adaptive behaviors in natural settings. To approach this goal in its most general form, it will be essential to monitor and manipulate both neural activity and behavioral variables, such as head and eye movements, while animals interact naturally with their environments. The availability of genetic tools to dissect neural circuitry (Luo et al., 2008) and to construct models of human disease (Götz and Ittner, 2008, Nestler and Hyman, 2010, Chesselet and Richter, 2011) has driven the emergence of the mouse as a key model organism in systems neuroscience (Carandini and Churchland, 2013). An increasingly wide array of technologies is available to measure and manipulate neural activity in mice (Voigts et al., 2008, Luo et al., 2008, Kim et al., 2017, Jun et al., 2017). However, detailed monitoring of behavior, especially in freely moving animals, remains a major challenge (Krakauer et al., 2017, Juavinett et al., 2018). To address this challenge, we developed a head-mounted camera system to measure eye position, pupil dilation, whisking, pinna movements, and other behavioral signals in freely moving mice, which we combined with head-movement monitoring and multichannel electrophysiology.

Despite the long-standing ability to record neural activity in unrestrained rodents (e.g., O’Keefe and Dostrovsky, 1971), many current studies of the neural basis of behavior have relied on awake but head-restrained animals (Carandini and Churchland, 2013, Juavinett et al., 2018). Head fixation enables tight control of sensory inputs, facilitates intracranial recording or imaging, and simplifies experimental manipulations that would be difficult in freely moving animals. However, results obtained in head-restrained animals may not generalize to more natural sensory and behavioral conditions. For example, the change in vestibular inputs following head fixation may have widespread effects throughout the brain (Rancz et al., 2015), and it is debated whether spatial navigation by head-fixed animals in virtual reality environments is comparable to spatial navigation in freely moving animals (Dombeck et al., 2007, Chen et al., 2013, Domnisoru et al., 2013, Schmidt-Hieber and Häusser, 2013, Aghajan et al., 2015, Minderer et al., 2016). While the level of experimental control and the availability of techniques for monitoring neural activity are more limited in studies of freely moving animals, such investigations have provided important insights into brain function during behavior that might not have been obtained in more constrained experimental settings; for instance, revealing cells that represent an animal’s spatial location and head direction (O’Keefe and Dostrovsky, 1971, Taube et al., 1990, Fyhn et al., 2004).

Detailed behavioral measurement in freely moving mice remains a major challenge because of the animal’s small size (the average weight of 8- to 16-week-old adult male C57BL/6J mice is ∼20–30 g; https://www.jax.org/). Externally mounted video cameras have been used to track aspects of gross locomotor behavior including gait (Machado et al., 2015) and posture (Hong et al., 2015, Wiltschko et al., 2015), and (in semi-stationary mice and when permitted by the camera angle) whisking (Voigts et al., 2008, Roy et al., 2011, Nashaat et al., 2017) and head and eye movements (Kretschmer et al., 2015, Kretschmer et al., 2017). However, the perspective of the external camera limits the potential for continuous measurement of whisking, pupil diameter, or eye position in actively exploring mice (although Payne and Raymond, 2017 have successfully monitored horizontal eye movements using a magnetic field approach).

The new miniaturized head-mounted tracking system reported here makes it possible to continuously monitor multiple behavioral variables, such as eye and pinna movements, whisking, eating, and licking, together with head movements, in combination with chronic neural recording from unrestrained mice. A recent study developed a head-mounted eye tracking system for the rat (Wallace et al., 2013). However, given the comparatively small size of the mouse, we required a system with a reduced weight and footprint. Moreover, the method used in rats relied on detection of reference points recorded by multiple video cameras and additional head-mounted LEDs to track orientation and movement of the head. Instead, we used inertial sensors to track the orientation and movements of the head (Mizell, 2003, Pasquet et al., 2016), simplifying the process of relating these variables to the camera outputs even under demanding natural conditions.

The system generates stable video output, leaves mouse behavior largely unchanged, and does not affect the quality of concomitant neural recordings. We demonstrate the potential of the system in a series of experiments in freely moving mice. First, we show that variables such as whisking frequency and pupil size vary systematically with behavioral state, and that these changes are correlated with neural activity, thereby generalizing results obtained in head-restrained mice to natural behaviors (Reimer et al., 2014, McGinley et al., 2015). Second, we demonstrate that a large fraction of variability in eye position in freely moving mice is explained by head movements, as has also been observed in rats (Wallace et al., 2013). We find systematic relationships between eye position and head orientation in freely moving mice, suggesting that mice stabilize their gaze with respect to the horizontal plane, even in the dark. Third, we demonstrate that neural activity in primary visual cortex (V1) is strongly modulated by head movements even in the absence of visual input. This effect does not depend on variability in eye movements and cannot be explained by whisking or locomotion. These results demonstrate how the new camera system can lead to novel insights into interactions between different behaviors and their relation with neural activity.

## Results

### A Miniature Head-Mounted Camera System for Freely Moving Mice

**Figure 1:**
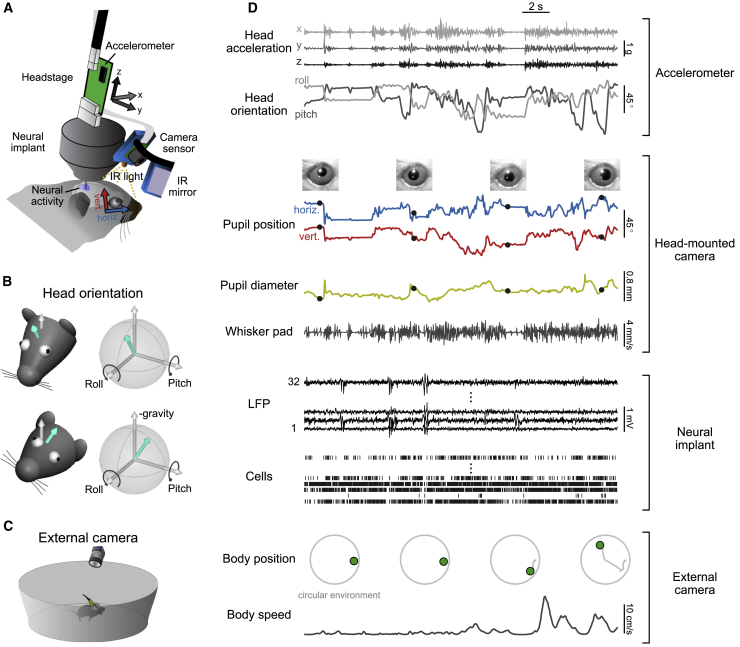
Simultaneous Measurement of Multiple Behavioral Variables and Neural Activity in a Freely Moving Mouse (A) Neural activity is recorded with a chronic tetrode implant; video data are simultaneously recorded using a miniature CMOS image sensor and an infrared (IR) mirror mounted on the implant with a custom holder. An IR light source on the camera holder illuminates the region of interest, which is imaged via the IR mirror. The mirror reflects only IR light, allowing visible light to pass through so the animal’s vision is not obstructed. Head motion and orientation are measured using an accelerometer integrated into the neural recording headstage. (B) Extraction of pitch and roll from low-pass filtered accelerometer signals. White arrow indicates direction opposite to gravity component. Turquoise arrow indicates orientation of vertical (ventral-dorsal) head axis. (C) A mouse freely explores its environment while wearing the head-mounted camera system. Absolute position is measured using external cameras. (D) Example traces of simultaneously recorded behavioral and neural data. Pictures of eye position in third row were acquired at times of dots on pupil position traces in the fourth row. See also Figures S1 and S2 and Video S1.

The head-mounted camera system (Figure 1A) consisted of a miniature CMOS image sensor with integrated video data cable, a custom 3D-printed holder for the image sensor, an infrared (IR) LED illumination source, and an IR mirror on a custom extension arm. The mirror reflected only IR light (reflectance > 95%) and allowed visible light to pass through (transmission > 80%), so it was visually transparent to the mouse (Peirson et al., 2018). The weight of the camera system including the image sensor was approximately 1.3 g (Figure S1; STAR Methods). We wrote custom software (STAR Methods) to synchronize video and neural data and to integrate video recordings with open-source systems for neural data acquisition (http://www.open-ephys.org). The camera system recorded video frames with an image resolution of 640×480 pixels at frame rates of up to 90 Hz (Figure S1); thus, video images could be aligned to neural data with a temporal precision of 11.1 ms.

The camera system was attached during each recording session to a miniature connector built into a chronically implanted custom tetrode drive with 8–16 individually movable tetrodes (based on an existing implant design; Voigts et al., 2013). Power to the IR LED was provided through the digital neural recording headstage, which was also attached to the implant for each recording session. The headstage board included an integrated 3-axis accelerometer to measure the movement and orientation of the animal’s head (Pasquet et al., 2016) (Figure 1B; see STAR Methods for measurement of rotational movements). The mouse freely explored a small circular environment, while body position was monitored using an external camera (Figure 1C). The combined system allowed the simultaneous measurement of pupil position, pupil dilation, whisker pad movement, head movement, head orientation, body position, and body speed together with neural activity (Figure 1D; Video S1). Signal quality of neural recordings was unaffected by the operation of the camera system (Figure S2; STAR Methods).

Video S1. Different Views with the Head-Mounted Camera, Related to Figure 1 Example views and behaviors that can be monitored using the head-mounted camera (exploration of objects, foraging, pinna movements, simultaneous monitoring of both eyes, simultaneous monitoring of eye/whisker movements and environment using two head-mounted cameras).

### Camera Images Remain Stable as the Mouse Moves

**Figure 2:**
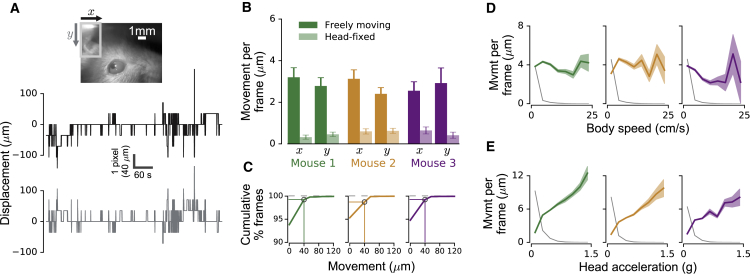
Image Stability during Movement (A) Camera view of the left eye (top) with inset showing reference for image registration (gray rectangle). Traces below show example frame-by-frame displacements of camera image in x- (middle) and y- (bottom) directions. (B) Average 2D inter-frame image movement (±SEM) for three mice, recorded while animals were either freely exploring a circular environment or head-fixed on a cylindrical treadmill. Number of freely moving and head-fixed recordings (10 min each): mouse 1, n = 55 and 22; mouse 2, n = 35 and 29; mouse 3, n = 14 and 14, respectively. (C) Cumulative distribution of inter-frame image movements. Note that image movement is zero for nearly 95% of frames. (D and E) Average inter-frame image movement (±SEM) as a function of body speed (D) or head acceleration (E), for three mice. Thin gray lines indicate relative frequency of body speed (D) or head acceleration (E). See also Video S2.

To measure the stability of video recordings from the head-mounted camera, we identified a rigid part of the implant visible in the image frame as a reference (gray outline in inset image in Figure 2A) and used motion registration (Dubbs et al., 2016) to determine the x- and y-displacement of the image in each frame, relative to the average image position across frames. When displacements occurred, they were typically on the order of a single pixel (40 μm; Figure 2A). The diameter of the mouse eye and pupil are approximately 3.4 mm (Sakatani and Isa, 2004) and 0.4–1.6 mm (McGinley et al., 2015), respectively. Thus, on average, camera image displacements in freely exploring mice were 1–2 orders of magnitude smaller than eye or pupil diameter. Moreover, average inter-frame image movement (i.e., change in 2D displacement between successive frames) was less than 4 μm in mice freely exploring a circular environment, compared to less than 1 μm in a control condition when the same animals were head-fixed on a cylindrical treadmill (Figure 2B; STAR Methods).

We also investigated the frequency with which image movements occurred in freely moving mice. Figure 2C shows the cumulative distribution of inter-frame image movements, after excluding frames in which the reference was occluded, e.g., during grooming (less than 0.6% of all frames; STAR Methods). In nearly 95% of analyzed frames, no image movement was observed. In 98%–99% of frames, the maximal shift was one pixel (40 μm; see marked points in Figure 2C).

Finally, we investigated whether image movement was related to mouse behavior. There was no evident relationship between average image movement per frame and body speed (Figure 2D). We also tested for a relationship with head acceleration (after removing the gravity component; Figure 1D; STAR Methods) and found an increase in image movement with stronger head accelerations, but these strong head movements were rare in all three mice (head acceleration magnitude less than 0.2 g for 95% of the recorded frames in all mice; Figure 2E). Moreover, even when mice made head movements with an acceleration magnitude of 1 g, the average image movement per frame did not exceed about 10 μm.

We conclude that the head-mounted camera system produced stable video recordings, even when mice were grooming or actively exploring objects in complex and enriched environments (Video S2).

Video S2. Video Image Stability, Related to Figure 2 Examples demonstrating stability of the head-mounted camera system during different behaviors (locomotion, grooming, running on wheel). Shown are raw video frames (i.e., without motion correction).

### Patterns of Behavior Are Minimally Disturbed by Camera System

**Figure 3:**
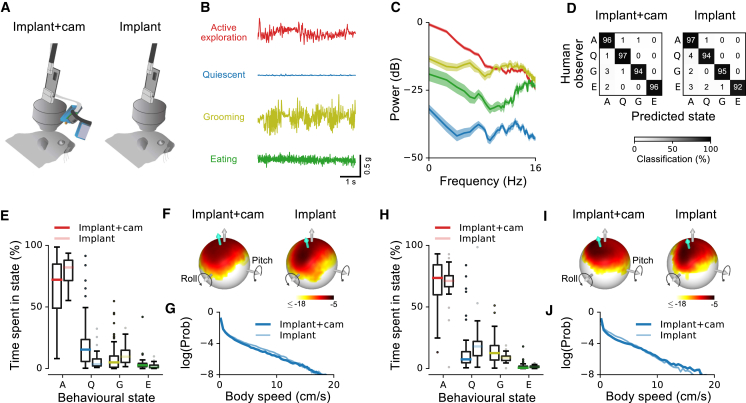
Impact of Head-Mounted Camera on Basic Mouse Behavior (A) Recordings were performed with (“Implant+cam”) and without (“Implant”) head-mounted camera system. (B) Example accelerometer traces for one motion axis recorded in different behavioral states. (C) Power spectra of accelerometer signals shown in (B), extracted from a 20-min recording. The different behavioral states can be reliably discriminated based on the power spectra. Shaded areas indicate SE. (D) Confusion matrix illustrating cross-validated classification performance of a semi-automatic state-segmentation algorithm based on head-mounted accelerometer signal spectra (“Predicted state”), compared to behavioral state classifications based on manual annotation of external video and other data (“Human observer”; STAR Methods). Left: mouse with implant and camera. Right: with implant only. (E) Distribution of proportions of time per session spent in different behavioral states for mouse 1. In each box plot, the central line indicates the median and the bottom and top edges of the box indicate the 25th and 75th percentiles, respectively. The whiskers extend to the most extreme data points not considered outliers (1.5 times the interquartile range) and the data points above or below the whiskers indicate outliers. All data were included in analyses. Dark and light colors of each hue indicate condition with and without camera, respectively. Number of sessions: implant+cam, n = 21; implant alone, n = 11. (F) Log-probability distribution of head orientation for the mouse in (E), with implant and camera (left) and with implant alone (right). Gray arrow indicates direction opposite gravity; turquoise arrow indicates mean head orientation. (G) Log-probability distribution of measured body speed for mouse 1. (H–J) The same as in (E)–(G) for mouse 2. Number of sessions: implant+cam, n = 18; implant alone, n = 11. See also Figure S3.

Previous work has shown that mice tolerate the tetrode implant with only minimal changes in natural behavior (Voigts et al., 2013). We wondered whether the additional weight and moment arm of the head-mounted camera system might alter gross locomotor and exploratory behaviors in our animals. We analyzed the head-mounted accelerometer signals obtained from two implanted mice with and without the camera attached, during repeated sessions of free exploration across more than 2 months. We developed a semi-automatic state-segmentation algorithm to segment the recordings into four behaviors (active exploration, quiescence, grooming, and eating) based on the short-term spectra of the accelerometer signals (STAR Methods; Figures 3B, 3C, and S3). We found that this approach more accurately matched human observer segmentation (with cross-validation) than approaches based on segmenting the time-domain accelerometer signals directly (Venkatraman et al., 2010, Dhawale et al., 2017) (Figures S3D and S3E). Cross-validated classifications of behavioral state using the spectra-based algorithm matched classifications by a human observer over 96% of the time both with and without the camera attached, with no significant difference in classification performance between the two conditions (Figure 3D, Fisher’s exact test, p = 0.40; Figure S3A, p = 0.13).

The successful semi-automated segmentation of behavioral states allowed us to objectively compare mouse behavior with and without the camera. Behavioral patterns varied from day to day (Figures S3B and S3C), but both animals spent the majority of time in the active exploration state in most sessions (Figures 3E and 3H). The proportion of time spent in each behavioral state depended in part on session number relative to the first recording (Figures S3B and S3C). However, we found no statistically significant differences between implant+cam and implant alone conditions in the proportion of time spent in each state for either mouse (permutation test, p = 0.07 for mouse 1, p = 0.12 for mouse 2; see STAR Methods for details). Each mouse divided its time similarly between the four behavioral states with and without the camera (Figures 3E and 3H).

Since the majority of time was spent in the active exploration state, we examined behavior in this state more closely, paying specific attention to head movements and body speed (Figures 3F and 3I). The addition of the camera produced a slight change in average head position (mouse 1, −7° pitch, +5° roll; mouse 2, −3° pitch, +5° roll), which was not statistically significant for either mouse (permutation tests; mouse 1, p = 0.41 pitch, p = 0.06 roll; mouse 2, p = 0.92 pitch, p = 0.37 roll). The camera also produced a small reduction in the standard deviation of head pitch, and a small increase in the SD of head roll (mouse 1, +3° pitch SD, −4° roll SD; mouse 2, +4° pitch SD, −6° roll SD), each statistically significant in one of the two mice (permutation tests; mouse 1, p = 0.04 pitch SD, p = 0.12 roll SD; mouse 2, p = 0.17 pitch SD, p = 0.04 roll SD), and even here the differences were relatively small (−11% for pitch in mouse 1 and +30% for roll in mouse 2). Distributions of body speed during active exploration were unaffected by the camera (Figures 3G and 3J; permutation test, p = 0.35 mouse 1, p = 0.39 mouse 2; STAR Methods). We conclude that active exploratory head and body movements were minimally affected by the presence of the head-mounted camera.

### Pupil Diameter and Whisking Correlate with Behavioral and Neural State in Freely Moving Mice

**Figure 4:**
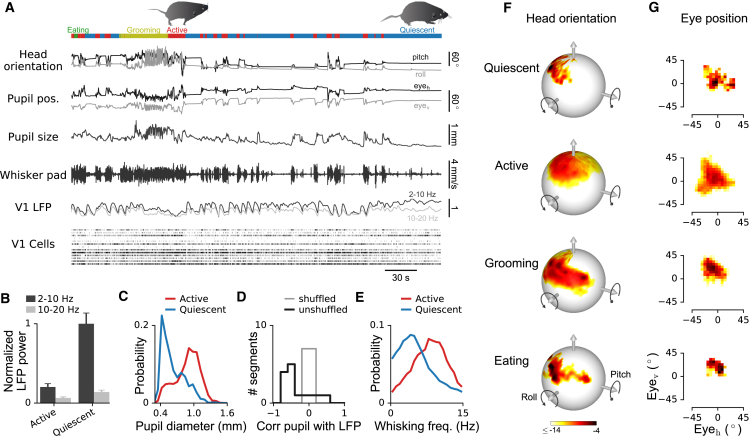
Continuous Monitoring of Behavioral and Neural Variables in Freely Moving Mice (A) Example traces of simultaneously measured behavioral and neural variables (6 min from a 40-min recording). Colored rectangles above traces indicate behavioral states assigned by the behavioral segmentation algorithm. (B) Low- (2–10 Hz) and high-frequency (10–20 Hz) LFP power in V1 in active and quiescent states (mean ± SEM). LFP power normalized by low-frequency power in quiescent state. (C) Distribution of pupil diameters in active and quiescent states. (D) Correlation coefficient between low-frequency (2–10 Hz) LFP power and pupil diameter during quiescent state. Only segments in which the head was still for at least 15 s were used for the analysis. (E) Distribution of whisker pad movement frequencies in active and quiescent states (30 Hz frame rates). (F) Log-probability distributions of head orientation in different behavioral states. (G) Log-probability distributions of simultaneously measured horizontal and vertical eye position in the same states. Same colorbar as in (F). See also Figures S4–S6 and Videos S3 and S4.

We next explored the capacity of the combined implant and camera system to identify correlations between behavioral and neural variables. Figure 4A shows a 6-min extract from a 40-min recording session of several behavioral and neural variables that included active and quiescent states, as well as grooming and eating (see Video S3 for a longer 10-min segment).

Video S3. Continuous Monitoring of Behavior, Related to Figure 4 Example segment (10 min, playback x 25) of the data shown in Figure 4.

Previous studies in head-restrained mice have indicated that low-frequency (2–10 Hz) local field potential (LFP) power in sensory cortex is significantly reduced when animals are actively exploring rather than quiescent (Poulet and Petersen, 2008, McGinley et al., 2015). Moreover, in head-fixed animals, pupil diameter is inversely related to low-frequency LFP power, and increased during active behavior and reduced during quiescence (Reimer et al., 2014, McGinley et al., 2015). We found that these relations also hold in V1 in freely moving mice (Figures 4B–4D). Normalized low-frequency LFP power was significantly lower in the active than quiescent state (Figure 4B; two-sample t test, p < 0.001), and the distribution of pupil diameters was shifted to larger values in the active state (Figure 4C; Wilcoxon rank-sum tests for difference in medians, p < 0.001). Low-frequency LFP power and pupil diameter were not only inversely affected by changes between active and quiescent behavioral states, but also negatively correlated in simultaneous recordings within the same behavioral state. We analyzed correlations between LFP power and pupil diameter for quiescent recording segments during which the mouse kept its head in a constant position for at least 15 s, to minimize fluctuations in pupil diameter from changes in eye illumination (see also STAR Methods and Figure S4 for more details on strategies for pupil tracking in freely moving mice). There was a strong negative correlation between pupil diameter and low-frequency LFP power in these recordings (Figure 4D; median correlation coefficient −0.44 versus 0 for shuffled data, Wilcoxon rank-sum test, p = 0.02).

Previous studies in head-restrained mice have also reported that the frequency of whisking is increased in the active compared to the quiescent behavioral state (Moore, 2004, Poulet and Petersen, 2008, Reimer et al., 2014). To examine whisking frequency in freely moving mice, we extracted whisker pad movements from the head-mounted camera images (see STAR Methods and Figure S5 for details) and observed an increased frequency of whisker pad movements in the active state (Figure 4E; Wilcoxon rank-sum test for difference in medians, p < 0.001), confirming previous findings in head-restrained mice. We also discovered an aspect of whisking behavior that has not, to our knowledge, been reported previously in head-restrained mice: sounds that were presented when the mouse was immobile reliably evoked whisker pad movements that were comparable in magnitude to whisker pad movements observed during active exploration (Figure S6; Video S4).

Video S4. Sound-Related Head and Whisker Movements, Related to Figure 4 An example segment of the data shown in Figures S6B and S6C.

The head-mounted camera system also enabled measurement and analysis of head movements and head-movement-related behavior, which cannot be studied in head-restrained animals. We measured the distributions of head orientation (Figure 4F) and eye position (Figure 4G) in four behavioral states (quiescent, active, grooming, and eating), by segmentation of behavioral data from continuous 40-min recording sessions (Figures 3A–3D). The distributions of both head orientation and eye position had wider spreads during active exploration than during quiescence (Figures 4F and 4G; permutation test, p < 0.001 for head pitch/roll and horizontal/vertical eye positions; STAR Methods). More specifically, the distributions in the quiescent state appeared to be dominated by particular combinations of head orientation and eye position that the mouse preferred at rest. In contrast, there was a different pattern during grooming: distinct modes of head orientation (which appeared to correspond to different grooming movements, e.g., forepaws over the nose and muzzle, strokes with the hindleg), combined with the same modal eye position (Figures 4F and 4G). Similarly, eye position remained relatively constant during eating, despite changes in head orientation. These observations indicate that head-eye coordination differs between behavioral states; eye-movement patterns are more restricted relative to head orientation during grooming and eating than during active exploration.

These results demonstrate that the head-mounted camera system enables detailed characterization of the relationship between multiple behavioral variables (such as head, eye, and whisker pad dynamics) and neural activity in freely behaving mice. In addition, it can help to reveal subtle aspects of natural behavior, such as sound-evoked whisking movements and differences in head-eye coordination between behavioral states.

### Eye Position Depends on Head Orientation in Freely Moving Mice

**Figure 5:**
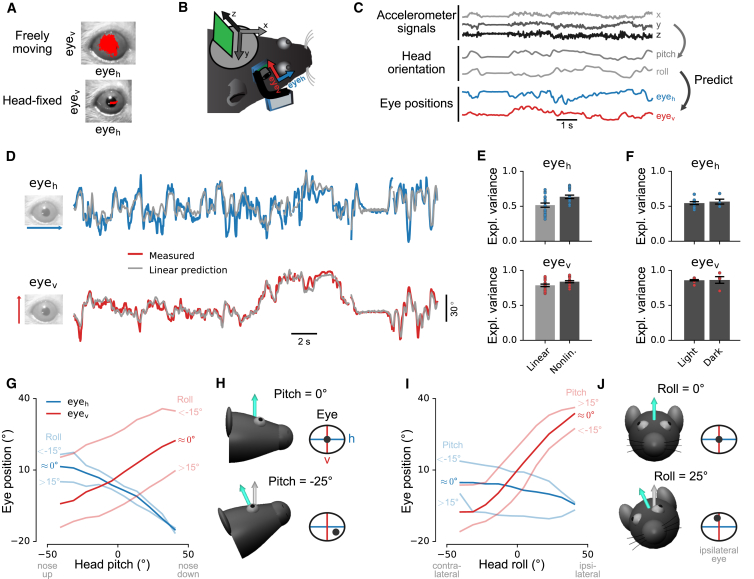
Systematic Relationships between Eye Position and Head Orientation in Freely Moving Mice (A) Measured eye positions (red dots) in a freely moving mouse (top) and in the same mouse during head fixation on a cylindrical treadmill (bottom). (B) Method for simultaneous recording of eye position and head acceleration. (C) Head orientation (pitch/roll) was computed from low-pass filtered head acceleration signals and was used to train models to predict eye position (arrows). (D) Measured eye positions compared to head-orientation-based predictions of a linear model. Model parameters were determined using training data different from the test data shown here. (E) Fraction of variance in eye position explained by head orientation, based on cross-validated predictions of linear (light gray) or nonlinear (dark gray) model. Top: horizontal eye position. Bottom: vertical eye position. Twenty recordings in 3 mice (n = 8,6,6 in mouse 1,2,3, respectively; 10 min each). (F) Fraction of variance in eye position explained by head orientation using the nonlinear model in light (n = 10 recordings) and dark (n = 4 recordings) conditions (all sessions from one mouse, 10 min each). (G) Horizontal (blue lines) and vertical eye position (red lines) as a function of head pitch. Dark and pale lines show interaction with head roll: “≈0°,” −15° < head roll < 15°; “<−15°,” head roll < −15°; “>15°,” head roll > 15°. (H) Illustration of systematic dependence of horizontal and vertical eye position on head pitch, for pitch = 0° (top) and pitch = −25° (bottom). Eye and eye coordinate system (h/v) rotates with head. (I and J) The same as in (G) and (H) but as a function of head roll, and with dark and pale lines showing interaction with head pitch. See also Figure S7 and Videos S5 and S6.

We wondered if the broader distribution of eye positions in actively exploring mice (Figures 4G and 5A, top) compared to quiescent mice (Figure 4G) or head-restrained mice moving on a cylindrical treadmill (Figure 5A, bottom; Video S5) was related to the larger range of head orientations during active exploration (Figure 4F). Previous results in head-restrained mice (Andreescu et al., 2005, Oommen and Stahl, 2008) and freely moving rats (Wallace et al., 2013) have suggested that average eye position varies with the orientation of the head. This is most evident in head-restrained, passively rotated mice, where eye position varies systematically with head pitch and roll (Oommen and Stahl, 2008). In order to examine the dependence in freely moving mice, we used head-mounted accelerometers to measure head orientation (pitch and roll) (Figures 5B and 5C; STAR Methods).

Video S5. Eye Movements in Freely Moving and Head-Fixed Mice, Related to Figure 5 Eye movements measured using the head-mounted camera system (left) and for the same mouse when it was head-fixed on a cylindrical treadmill (right). No stimuli or visual feedback were provided during the head-fixed recording.

First, we examined the accuracy with which head pitch and roll predicted eye position (Figure 5C). Regression models based on these two variables were able to capture a large fraction of the variation in horizontal and vertical eye positions (Figures 5D and 5E; see also STAR Methods and Video S6). For a simple linear model, cross-validated explained variance between measured and predicted eye position was 52% for horizontal and 79% for vertical eye position; for a nonlinear model (STAR Methods), explained variance was 64% and 84%, respectively (Figure 5E). Results were consistent over multiple months within and across mice, as indicated by the stability of regression model weights (Figure S7A). Explained variances were comparable in lit and dark environments (Figure 5F; see STAR Methods for details), indicating that this effect of head orientation on eye position was driven by vestibular input or efferent copy signals rather than visual input (Andreescu et al., 2005, Oommen and Stahl, 2008).

Video S6. Prediction of Eye Movements, Related to Figure 5 Measured (red) and predicted (blue) eye position of a freely exploring mouse. Predictions based on a nonlinear model and head pitch/roll as shown in Figures 5E and 5F.

Model predictions of eye position based on head pitch and roll were significantly more accurate for vertical than horizontal eye position (Figure 5E; Wilcoxon signed-rank test, p = 2⋅10^−6^ for linear model, p = 1⋅10^−5^ for nonlinear model). We wondered if the horizontal eye position might be more affected than the vertical by correlated movements across the two eyes independent of head orientation and used a dual-camera system to monitor both eyes simultaneously (Figures S7C and S7D; Video S1). We then trained predictive models on data from each eye individually and found that the interocular error correlation (the correlation between variability in eye position not explained by pitch and roll of both eyes) was significantly stronger for horizontal than vertical eye position (*cc* = 0.72 horizontal, *cc* = 0.11 vertical; Wilcoxon signed-rank text, p = 0.002; n = 10 recordings in one mouse, 10 min each).

We further asked if rotational head movements around the gravity axis (yaw), which are not well captured by the head-mounted linear accelerometer, might also account for the apparently weaker dependence of horizontal than vertical eye position on head orientation. To test this, we added a gyroscope to the implant (STAR Methods). Including rotations about the yaw axis increased the variance explained by the linear and nonlinear models by approximately 0.10 in horizontal and 0.02 in vertical eye position (Figure S7E), confirming some contribution of head yaw movements to prediction of horizontal eye position. The linear weights associated with the yaw signal were also remarkably similar across recordings (Figure S7B). In three recordings in the mouse with dual-camera implants and gyroscope, we found that interocular error correlation in the horizontal direction increased from 0.72 (head pitch/roll only) to 0.78 (including yaw as covariate) with no change in interocular error correlation in the vertical direction (0.12). Thus, coupled variation of eye position unexplained by orientation or rotation occurs primarily in the horizontal direction and may be caused by correlated eye movements not dependent on head movement; for example, during resetting eye movements (van Alphen et al., 2001, Stahl, 2004) or continuous drift toward a resting eye position (van Alphen et al., 2001, Wallace et al., 2013).

Figures 5G and 5I summarize the effects of head orientation on eye position. Both horizontal and vertical eye position varied systematically (and approximately linearly) with head pitch (Figure 5G) while vertical eye position was primarily affected by head roll (Figure 5I), consistent with reports in head-fixed mice (Oommen and Stahl, 2008) and freely moving rats (Wallace et al., 2013). Predictions of horizontal eye position were further improved by incorporating head yaw signals from a head-mounted gyroscope (Figure S7E). These results indicate that eye position is closely linked to head orientation in freely moving mice, even in the dark and even when the animals are exploring objects in enriched environments (Video S6).

### Rapid Eye Movements Are Strongly Linked to Head Movements in Freely Moving Mice

**Figure 6:**
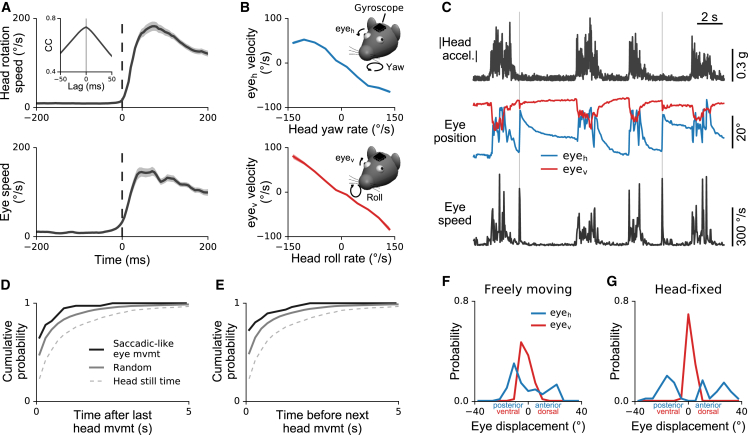
Coupling between Eye Movements and Head Movements (A) Dynamics of head movement (top) and eye movement (bottom) during head movement initiation. Head rotation speed was measured using a gyroscope attached to the implant; eye speed computed from pupil positions. Traces were aligned to the onset of head movement (rotational speed ≥ 15°/s with at least 0.5 s of no movement before onset). Plots show mean ± SEM for n = 160 head movement events in one mouse, recorded in 14 different 10-min sessions across more than 4 months. Inset shows average cross-correlation between head and eye speed; note peak at zero time lag. (B) Top: average horizontal eye velocity as a function of head velocity about the yaw axis. Directions as shown in inset. Bottom: average vertical eye velocity as a function of head velocity about the roll axis. In both directions, eye movements counteract head rotations. Plots show mean ± SEM (smaller than line width). Same dataset as in (A). (C) Rapid eye movements occurring in the absence of head movements. Example traces showing magnitude of head acceleration computed from accelerometer signals (top), horizontal/vertical eye positions (middle), and eye speed computed from eye positions (bottom). Saccade-like eye movements occurring in the absence of head movements (thin vertical lines) were identified by detecting eye movements with peak eye speed > 250°/s, which occurred when head movements were below a fixed threshold (0.0625 g). (D) Cumulative probability of the time between detected saccade-like eye movements and the preceding head movement (solid dark line). For comparison, cumulative probability is also shown for simulated data (solid gray line) with the same saccade-like eye movement rate but with saccades occurring at random times within the recorded head-still times (dashed line). Saccade-like eye movements were significantly more likely to occur soon after a head movement than would be expected by chance (Kolmogorov-Smirnov test, p = 3.5⋅10^−8^). Same dataset as in Figure 5 (20 recordings in 3 mice, 10 min each). (E) Same as in (D) but for the time between saccade-like eye movements and subsequent head movements. Saccade-like eye movements were significantly more likely to occur just before a head movement than would be expected by chance (Kolmogorov-Smirnov test, p = 2.2⋅10^−7^). (F) Changes in horizontal and vertical eye position from 20 ms before to 20 ms after the time of peak speed in saccade-like eye movements. Saccade-like eye movements tend to be larger horizontally than vertically. Same dataset as in (D) and (E). (G) Same as in (F) but for mice head-fixed on a cylindrical treadmill (4 recordings in 2 mice, 10 min each).

We next investigated the relationship between eye and head dynamics. Angular head velocity was measured with the head-mounted gyroscope described above. Eye speed measurements taken around the time of increases in head rotation speed revealed a close correspondence between the temporal profiles of eye movements and head movements (Figure 6A), with the eye typically moving in the opposite direction to the head (Figure 6B). These results are consistent with the observed dependence of eye position on head orientation (Figures 5G–5J) and with the expected effects of the vestibulo-ocular reflex (VOR; Stahl, 2004).

Despite this close overall coupling between head and eye movements, saccade-like (>250°/s; see, for example, Sakatani and Isa, 2007) eye movements were occasionally observed in the absence of head movements (Figure 6C), occurring at an average rate of 0.044/s during head-still times. Moreover, these saccade-like eye movements were not uniformly distributed during head-still times, but were significantly more likely to occur right before or after a head movement (Figures 6D and 6E). Saccade-like eye movements were qualitatively similar with and without head restraint. Figures 6F and 6G show the distribution of eye displacements in the horizontal and vertical direction for saccade-like eye movements in freely moving and head-fixed mice, respectively. Interestingly, the largest eye displacements in freely moving mice were observed in the horizontal direction, consistent with the pattern in head-fixed animals. In freely moving mice, however, the range of horizontal eye displacements was slightly reduced (median movement magnitude 9.9° and 17.7°, respectively; Wilcoxon test, p < 3⋅10^−8^), perhaps reflecting greater reliance on head movements for gaze shifts.

We conclude that eye movements are generally closely coupled to head movements in freely moving mice. Occasionally, the eye moves in the absence of head movement—but this typically happens just before or after the head moves. Together with the previous observation that average eye position is closely linked to head orientation even during active exploration, these results indicate strong interactions between eye and head movements at both fast and slow timescales in freely moving mice.

### Visual Cortex Activity Is Modulated by Head Movements in the Dark

**Figure 7:**
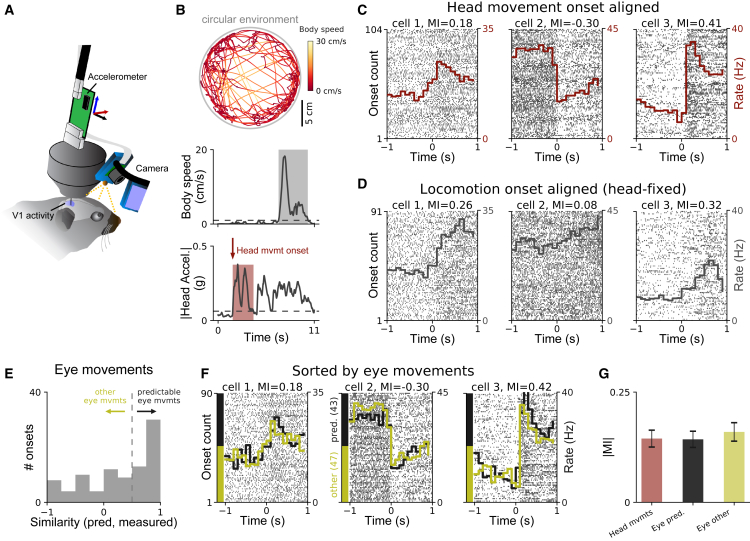
Head Movement-Related Modulation of Firing in Visual Cortex (A) Chronic tetrode implant, head-mounted camera system, and head-mounted accelerometer were used to record neural activity in primary visual cortex (V1), eye positions, whisker pad movements, and head movements while mice explored a circular environment in the dark. (B) Top: body position and speed were tracked using an external camera. Middle: periods when body speed exceeded 1 cm/s (gray rectangle) were excluded from consideration in order to focus on head movements occurring without locomotion. Bottom: a head movement episode (red area) was defined as a period when body speed was less than 1 cm/s and head movement was above threshold (dashed line) following at least 0.5 s below threshold (before head movement onset). (C) Raster plots for three simultaneously recorded V1 cells, showing spike times relative to head movement onset. Rasters are displayed vertically according to onset count (i.e., time order) within recording (left axis). Red histograms show the average spike rate across all extracted onsets (right axis). For all three cells, firing rate was significantly modulated by head movement (Wilcoxon signed-rank test, pre versus post movement onset; p < 0.001). (D) Raster plots for the same cells as in (C) but aligned to locomotion onset (threshold 1 cm/s) for mouse head-fixed on a cylindrical treadmill. (E) Division of eye movement onsets into those well-predicted by a model based on head orientation (cosine similarity ≥ 0.5) and other eye movements (cosine similarity < 0.5). (F) Raster plots and firing rate histograms for the same three cells as in (C), for the two types of eye movement onsets shown in (E). Spike train data same as in (C) but including only head-movement onset events for which the eye movement could be reliably extracted. Rasters are grouped vertically by eye movement onset type as indicated by colored y axis bars (“predictable,” black; “other,” yellow). Spike rate histograms shown overlaid using same color convention. (G) Summary of modulation indices (MI; see text) for V1 activity when aligned to head movement onsets, eye movement onsets that were predictable from head acceleration, or eye movement onsets that were not predictable from head orientation. Plot shows mean ± SEM across 16 recordings (20–40 min each) in 3 mice (74 cells with at least 2 spikes per second). See also Figure S8.

When combined with an implanted neural recording device, the head-mounted camera and motion sensor make it possible to investigate how brain activity is modulated during natural movements in freely moving mice. Previous work has indicated that locomotion modulates visual cortical activity in head-restrained mice (Niell and Stryker, 2010, Saleem et al., 2013, Fu et al., 2014, Reimer et al., 2014). We wondered whether head movements would evoke distinct patterns of activity in V1, given that V1 receives substantial vestibular input accompanying eye movements (Rancz et al., 2015, Vélez-Fort et al., 2018) along with inputs from many other non-primary sensory areas (Leinweber et al., 2017). We measured pupil, whisker pad, and head movements along with neural activity in single cells in V1 while animals freely explored a circular environment (Figure 7A) in the dark (to exclude the possibility of uncontrolled visual inputs during head movement). We tracked the body of the mouse with an external camera and excluded periods of gross body movement (≥1 cm/s) to analyze head movements that were not accompanied by locomotion (Figure 7B; STAR Methods).

Activity was tightly locked to head-movement onsets in many visual cortical cells. In total, 55% (41/74) of V1 cells were significantly modulated by head movement (see STAR Methods for details on spike sorting and data extraction). We observed both increases and decreases in firing rate even for simultaneously recorded cells (Figure 7C). To quantify the movement-related response modulation of individual cells, we computed a modulation index MI = (Post − Pre)/(Post + Pre), where Post and Pre are the mean firing rates for 1 s after and before movement onset, respectively. As shown for the three simultaneously recorded cells in Figures 7C and 7D, V1 response modulation at the onset of head movements without locomotion in unrestrained mice could be similar to or different from V1 response modulation at the onset of locomotion in the same animals head-fixed on a cylindrical treadmill. There was no significant correlation between the firing patterns of 74 V1 cells recorded in both conditions in 3 different mice (Wald test, p = 0.18; Figure S8A). This observation suggests that head movements can affect firing rates of visual cortex neurons independently of locomotion.

Head movements were tightly coupled to eye movements in freely moving mice (Figure 6A). We wondered whether the potential relevance of the eye movement to gaze stabilization mattered to the modulation of V1 activity we observed in the dark. We extracted the first eye movement in the period around head movement onset by measuring optical flow of the pupil edges in the dark (STAR Methods; Figures S8G–S8I). We then separated movement onsets into those in which the initial eye movement was well predicted by the head accelerometer data according to the models described above, and those in which the movement was less predictable (cosine similarity between measured and predicted eye movement directions ≥ 0.5 or < 0.5, respectively; approximately half of the movement onsets fell into each group; Figure 7E).

Sorting data by whether or not the eye movements were consistent with the accompanying head movement did not reveal any systematic differences in neural activity (Figure 7F), and absolute modulation indices around head movement onsets did not depend on whether the eyes moved predictably (Figure 7G; p = 0.8, “eye predictable” versus “eye other”; p = 0.06, all onsets versus “predictable”; p = 0.15, all onsets versus “other”; Wilcoxon signed-rank tests, n = 37 cells with at least 20 movement events of each type; see also Figure S8C). Thus, modulation of V1 responses by joint head and eye movements in the dark did not appear to depend on the extent to which the eye movements contributed to gaze stabilization.

We next asked whether whisker movements differentially affected modulation of neural responses in V1. While whisking often accompanied head movement, it was not as strongly synchronized as were eye movements. V1 neurons showed less firing-rate modulation aligned to the onset of whisking than to head movement (Wilcoxon signed rank test, p = 0.001). When aligned to whisking events that were not accompanied by head movement, V1 modulation was smaller still (Wilcoxon signed-rank test, p < 3⋅10^−6^; Figures S8D–S8F). Thus, head movements modulated V1 activity more strongly than whisking movements in most recorded cells.

We conclude that head movements modulate V1 activity in freely moving mice, even in the dark and in the absence of locomotion. Moreover, while head, eye, and whisker movements are coupled in freely moving mice, modulation of V1 activity does not depend on the coordination of head and eye movement direction, and cannot be fully explained in terms of whisking alone.

## Discussion

The mouse is a prominent animal model in neuroscience, but behavioral monitoring in freely moving mice has been limited by the absence of video tracking methods in head-centered coordinates. To overcome this limitation, we developed a miniature head-mounted video camera system and combined it with movement sensors to monitor multiple behavioral variables including pupil size and eye position as well as head, whisker pad, and body movements, and integrated it with a chronic multielectrode implant to record neural activity in freely moving animals (Figure 1; Video S1). The camera system is stable, enabling precise and continuous monitoring of behavioral variables and minimizing the amount of postprocessing required to extract the variables of interest. Inter-frame image movement was less than 1 pixel (corresponding to about 40 μm) in about 99% of all video images, even when the mice were grooming, exploring complex environments, or interacting with objects in the environment (Figure 2; Video S2). Crucially, mouse behavior was similar with and without the camera system (Figure 3), allowing accurate monitoring of pupil size, eye position, whisking, and other variables during natural behaviors. The operation of the camera system did not affect the quality of simultaneous electrophysiological recordings.

This new head-mounted camera system significantly expands the range of scientific questions that can be addressed in freely moving mice. Ethological studies could reveal the precise characteristics of behavior such as eye movements, whisking, and other motor outputs. Sensory neuroscientists could use the system to validate experimental results obtained under conditions of head or body restraint—while directly studying sensory processing under more natural conditions. Studies of non-sensory brain areas, including associative and motor areas, could identify sources of behavioral variability that drive neural activity but have been previously hard to measure. Mouse models of disease could be examined to establish or to exclude deficits in eye movements, whisking, or other motor outputs.

Here we have shown that the head-mounted camera system can provide new insights into the relationships between eye, head, and whisking movements and neural activity in freely moving mice. In many animals, eye and head movements are intimately related and both are used for orienting gaze toward salient objects (Land, 2015). However, very little is known about their coordination in mice, even though this information could provide important general insights into how non-foveate animals use vision during natural behavior. We observed prominent changes in the distributions of both head orientation and eye position in different behavioral states in freely moving mice (Figure 4). When we quantified this relationship using predictive models, we discovered that a large fraction of the variation in eye position could be predicted from head orientation, consistent with findings from a previous study in the freely moving rat (Wallace et al., 2013). Our results suggest that freely moving mice stabilize their gaze relative to the horizontal plane. Moreover, our data show that this gaze stabilization does not only happen on average but also at a fine temporal resolution (Figure 5; Video S6), and therefore may play an important role in mouse vision. We also found that the systematic relationships between eye position and head orientation were preserved across months, across mice, and in the dark as well as the light, suggesting that head-orientation-related changes in eye position are driven by vestibular rather than visual input (Oommen and Stahl, 2008).

While models based on head orientation and rotational head movements were able to explain most variation in eye position, particularly in the vertical direction, there was still considerable unexplained variance in the horizontal direction (about 10%–50%). By using two head-mounted cameras, we found that horizontal eye positions not explained by head orientation were strongly correlated across both eyes, even after taking into account rotational movements of the head. Whether these correlations resulted from resetting eye movements not locked to head movements (e.g., van Alphen et al., 2001) or active shifts in gaze will need to be determined in future work. Most of the present experiments were done in a circular environment without salient visual objects. However, in enriched environments it appeared that mice did not orient their eyes toward objects even when they actively explored them (Video S1). Moreover, even saccade-like eye movements occurring without a coincident head movement were significantly more likely to occur just before or just after a head movement than would have been expected by chance (Figure 6). Future experiments might use the camera system to investigate whether freely moving mice encountering highly salient or moving visual objects produce more eye movements that are not coupled to head orientation or head movements. More generally, the camera system could be used to resolve ongoing debates about how non-foveate animals with laterally positioned eyes coordinate the position and overlap of the two eyes during different behavioral tasks (Wallace et al., 2013, Meister and Cox, 2013). Monitoring not only the eyes but also the environment using a head-mounted camera facing outward without IR mirror (Video S1) could help to clarify the link between head and eye movements and visual inputs.

We also demonstrated how the camera system can be combined with motion sensors and chronic neural recording devices to discover new relationships between motor-related variables and neural activity in the visual cortex. About 55% of V1 cells were modulated by head movements in the absence of locomotion, and of any visual input. Both enhancement and suppression of firing were seen, even for cells recorded at the same time (Figure 7). These results were not explained by variations in eye movements or whisking. Recent work has demonstrated that locomotion can modulate activity in sensory cortex (Niell and Stryker, 2010, Schneider et al., 2014). For example, in mouse primary visual cortex, neural responses are generally enhanced when head-fixed animals run on a treadmill compared to when they are stationary (Niell and Stryker, 2010); in contrast, in primary auditory cortex, neural responses are typically suppressed by locomotion (Schneider et al., 2014). We measured changes in neural activity in primary visual cortex either during head movements in the absence of locomotion when the mouse was freely moving, or during locomotion when the mouse was head-fixed on a cylindrical treadmill. We found that the directions of modulation in the same V1 neuron could be different for locomotion-related and head-movement-related responses, and that there was no significant correlation between the two types of movements. These results demonstrate that modulation of early sensory cortical areas by motor outputs is both more general (i.e., occurring for many forms of movement) and more specific (i.e., manifested differently for different forms of movement) than previously thought.

We were not able to disentangle the effects of head movements and locomotion on V1 activity in freely moving mice because our unrestrained animals rarely ran, possibly because the environment they explored was relatively small. Moreover, although head movements often occurred without locomotion in the unrestrained animals, locomotion rarely occurred without head movements. Further studies might isolate locomotion modulation in freely moving animals by training mice to run across narrow corridors to minimize head movement, an approach that could also reveal possible effects of movement restriction in head-fixed conditions (e.g., see Minderer et al., 2016). Future work will also be needed to identify whether the movement-related signals are used for suppression of sensory coding during self-generated movement (e.g., saccadic suppression; Duffy and Burchfiel, 1975), for the computation of the mismatch between sensory input and expected input (Keller et al., 2012), or for the integration of sensory inputs with signals related to spatial navigation (Saleem et al., 2013). We anticipate that important progress can be made by combining our method with new tools for virtual reality in freely moving animals (Stowers et al., 2017, Del Grosso et al., 2017) to provide both detailed behavioral and stimulus control.

The new system is open source and we provide all required software and design files. To our knowledge, this is the first open-source head-mounted video tracking system for small laboratory animals. The system uses widely available components (e.g., camera sensor, single-board computer, and connectors) or 3D-printable parts (camera holder), and the total cost is low (see parts list), which should further promote its adoption. Moreover, the system could be easily adapted for use in larger animals, such as rats, ferrets, and monkeys. At the moment the system is tethered, but especially in larger animals it is possible to add batteries to power the system so it can be used in conjunction with wireless recording methods (Fan et al., 2011, Yin et al., 2014). In the mouse, a major challenge remains the weight of the combination of headposts, cameras, parts for neural recordings, batteries, and wireless transmitters, but technical developments in miniaturizing all these components might make entirely wireless head-mounted neural recording and behavioral monitoring systems feasible in the near future. Furthermore, the system is modular and could be integrated with alternative methods for recording neural activity, such as high-density silicon probes (Jun et al., 2017) or head-mounted fluorescence microscopes (Zong et al., 2017), and/or combined with technologies for optogenetic manipulation of neural activity during behavioral monitoring (Wu et al., 2015).

Because the position of the camera and mirror can easily be customized, the view can be modified to include other variables of interest. For example, a small modification to the arm holding the mirror is sufficient to provide a detailed image of the pinna (Video S1) to provide insights into how pinna movement contributes to the processing of incoming sounds, e.g., during sound localization, in freely moving animals. The camera could also be used to monitor the movement of single whiskers in head-centered coordinates, as opposed to the whisker pad movements tracked in the current study, without the need for external tracking cameras, computation of absolute position in space, or attachment of markers to single whiskers (Voigts et al., 2008, Roy et al., 2011, Nashaat et al., 2017). Finally, the camera system can also be used to capture images of the nose, mouth, and/or paws, to monitor how mice interact with their environment when they explore novel objects (see Video S1 for a mouse interacting with Lego and foraging) and during social behaviors such as mating and fighting. Thus, the system has the potential to greatly increase the range and scope of experimental questions that can be addressed about natural behaviors in freely moving mice and other small laboratory animals.

## STAR★Methods

### Key Resources Table

**Table:** 

REAGENT or RESOURCE	SOURCE	IDENTIFIER
**Experimental Models: Organisms/Strains**		

Mouse: C57BL/6J	Charles River	Strain code: 027
Mouse: CBA/Ca	Charles River	Strain code: 609

**Software and Algorithms**		

Python 2.7	https://www.python.org/	RRID: SCR_008394
Open Ephys plugin-GUI	http://www.open-ephys.org	https://github.com/open-ephys/plugin-GUI
SpikeSort	Sahani, 1999	N/A
Fiji	http://fiji.sc	RRID: SCR_002285
moco	Dubbs et al., 2016	https://github.com/NTCColumbia/moco
R	http://www.r-project.org/	RRID: SCR_001905
Custom camera software	This paper	http://www.gatsby.ucl.ac.uk/resources/mousecam/
Data extraction and analysis code (including pupil fitting and behavioral segmentation code)	This paper	http://www.gatsby.ucl.ac.uk/resources/mousecam/
OpenSCAD	http://www.openscad.org/	N/A

**Other**		

Flexdrive	http://www.open-ephys.org	http://www.open-ephys.org/flexdrive/
Microwire (17 μm, platinum iridium)	California Wire Company	Cat#100167
NanoZ plating equipment	Multichannel Systems	nanoZ, https://www.multichannelsystems.com/products/nanoz
Open Ephys acquisition board	Open Ephys	http://www.open-ephys.org/acq-board/
Intan RHD2132 neural recording headstage	Intan Technologies	Cat#C3324
Ultra Thin RHD2000 SPI cable	Intan Technologies	Cat#C3216
Camera system design files (scad/stl) / Assembly instructions	This paper	http://www.gatsby.ucl.ac.uk/resources/mousecam/
CMOS Camera sensor	Adafruit	Cat#1937
NIR-Blocking Filter Calflex-X, 25mm x 25mm	Qioptiq	Cat#G380227033
Hot Mirror, 20mm x 20mm	Edmund Optics	Cat#62-627
Thin wall steel cannula 21 G	Coopers Needle Works	N/A
Miniature connectors (NSD-18-DD-GS, NPD-18-DD-GS)	Omnetics	Cat#A79007-001, Cat#A79006-001
Miniature infrared LED	Vishay	Cat#VSMB2943GX01
Multicomp resistor, 100 – 180 Ohm, metric package size 3216	Farnell	N/A
RS Pro Male and Female Solder D-sub Connector Contact	RS Components	Cat#481-493, Cat#481-500
Raspberry Pi 3 Model B+	Farnell	Cat#2842228
External tracking cameras	The Imaging Source	Cat#DMK23UV024

### Contact for Reagent and Resource Sharing

Further information and requests for resources and reagents should be directed to and will be fulfilled by the corresponding authors, Arne F. Meyer (arne@gastsby.ucl.ac.uk), Jasper Poort (j.poort@ucl.ac.uk), and Jennifer F. Linden (j.linden@ucl.ac.uk).

### Experimental Model and Subject Details

#### Animals

Experiments were performed on male C57BL/6J mice (Charles River) for visual cortex recordings and male C57BL/6J and CBA/Ca mice (Charles River) for the sound experiment. Mice aged 58–65 days were implanted with chronic implants for neural recordings. After surgical implantation, mice were individually housed on a 12-h reversed light-dark cycle (lights off at 12.00 noon). Water and food were available *ad libitum*. All experiments were performed in healthy mice that were not used for any previous procedures. All experimental procedures were carried out in accordance with the institutional animal welfare guidelines and a UK Home Office Project Licence approved under the United Kingdom Animals (Scientific Procedures) Act of 1986.

### Method Details

#### Surgical procedures

For chronic implants, we used custom tetrode hyperdrives with 8–16 individually movable tetrodes, constructed according to a published design (Voigts et al., 2013). Tetrodes were made from HM-L coated 90% platinum/10% iridium 17 μm diameter wire (California Fine Wire, USA). A miniature male connector (NPD-18-DD-GS, Omnetics, USA) was attached to the front of the drive body (see “Construction of the camera system”) for connection of the camera system during behavioral experiments.

Mice aged 58–65 days were anaesthetized with 1%–2% isoflurane and injected with analgesia (Carprofen, 5 mg/kg IP). Ophthalmic ointment (Alcon, UK) was applied to the eyes, and sterile saline (0.1 ml) injected SC as needed to maintain hydration. A circular piece of dorsal scalp was removed and the underlying skull was cleaned and dried. A custom machined aluminum head-plate was then cemented onto the skull using dental adhesive (Superbond C&B, Sun Medical, Japan). A small craniotomy was made over the left primary visual cortex (V1) (2.5 mm lateral, 1 mm anterior to the transverse sinus). The tetrode drive was positioned above the craniotomy and fixed to the skull with dental adhesive. A pinhole craniotomy was made above the right prefrontal cortex contralateral to the tetrode implant for the ground screw (000-120 × 1/16, Antrin Miniature Specialties, USA). The ground screw and implant were then secured with more dental adhesive and dental cement (Simplex Rapid, Kemdent, UK). Mice were allowed to recover from surgery for at least five days before experiments began.

#### Neural recordings in head-fixed and freely moving mice

All experiments were conducted in a custom double-walled sound-shielded anechoic chamber. Animals became accustomed to handling and gentle restraint over two to three days, before they were head-fixed and placed on a custom styrofoam cylinder (20 cm diameter, on a ball-bearing mounted axis). After animals were head-fixed the headstage was connected to the implant and the camera holder was connected to the miniature connector on the outside of the implant, together with two cables from the headstage which provided power to the IR light-emitting diode (IR LED).

We confirmed that each tetrode recording site was in monocular V1 by presenting stimuli on a screen contralateral to the implant side and identifying the approximate receptive field position of recorded cells as described previously (Poort et al., 2015). Luminance of visual stimuli was calibrated using a luminance meter (LS-100, Konica Minolta, Japan). Running speed on the cylinder was detected with a rotary encoder (1024 steps per rotation, Kübler, Germany) and single steps were extracted using a microcontroller (Arduino Uno, Farnell, UK), sent to the recording system as transistor–transistor logic (TTL) pulses and recorded along with neural data.

For experiments in freely moving mice, the implant was gently held while allowing the mouse to walk or run on a running wheel, and headstage and camera system were connected as for the head-restrained experiments. The animal was then released into a circular environment for experiments in the freely moving condition. Two different circular environments were used. The first environment (diameter 30 cm) consisted of white plastic material. Eight LED lights (ULT300, Digital Daffodil, UK) combined with custom cut light diffuser sheets (Perspex, UK) were used to provide homogeneous lighting which facilitated tracking of the eye (see “Extraction of pupil positions from camera images”). For the sound experiment (Figure S6) a loudspeaker was mounted 1 m above the center of the environment (see “Sound presentation”). The second environment (diameter 22 cm) consisted of black plastic materal with a semi-transparent perspex floor to allow reliable tracking of body position using an external camera from below (see “Analysis of head movement onsets”). This second environment was used to perform recordings in the dark (Figure 7).

Neural activity was recorded with a 32-channel Intan RHD 2132 amplifier board (hardware bandpass filtering between 1.1 and 7603.8 Hz; Intan Technologies, USA) connected to an open-ephys acquisition board (Open Ephys) via a flexible serial peripheral interface cable (“Ultra Thin RHD2000 SPI cable,” Intan Technologies, USA). Data were sampled at 30 kHz and saved to disk for offline analysis.

#### Electrophysiological data analysis

Electrophysiogical recordings were analyzed offline using Bayesian spike-sorting techniques (Sahani, 1999). To detect action potentials the common median reference was subtracted across channels (Rolston et al., 2009) with subsequent high-pass filtering with a cutoff of 600 Hz, and action potentials were detected by finding time points exceeding 3.5 times the standard deviation of the noise. Action potentials were automatically clustered. Single units or small clusters of neurons were accepted only if the spike-sorter reported both false-negative and false-positive rates below 5%. Clustered units were verified manually and units were classified as single-unit (SU) if fewer than 0.5% of the spikes occurred within the typical refractory period of a cortical neuron (≤2 ms). All other units were deemed multi-units (MUs).

The effect of the head-mounted camera system on neural recording quality was assessed using raw broadband signals and spike units (158 SUs and 11 MUs). The power spectral density (PSD) of broadband signals was estimated using Welch’s method with a 2 s long Hann window and 1 s overlap. For each condition, the PSD of all electrode channels was computed separately and the log-scaled PSDs averaged afterward to yield a single estimate of the PSD (Figure S2C). To quantify the difference across all recordings, we computed the PSD ratio between segments with camera on and off (10 min each) recorded during the same session without disconnecting the neural recording headstage (Figures S2D–S2F). The order of the two conditions was balanced across sessions to reduce potential effects of behavioral changes during each session (e.g., mice typically explored the environment more during the early part of the recording). Within-condition variability for the implant-only condition was estimated by computing the standard deviation of PSD ratios for different non-overlapping 60 s segments from the same recording. The signal-to-noise ratio (SNR) between spikes and high-pass filtered electrode signals (Figure S2G) was computed as the power of the electrode channel of each tetrode with maximum depolarization, and the noise power extracted from electrode signals between spikes (with a 2 ms margin around spikes). All data recorded during the same session were spike sorted together to avoid the need to manually register spike clusters between conditions.

To compute the power in the local field potential (LFP), raw traces were first bandpass filtered at 2 – 10 Hz (low-frequency LFP in Figure 4) or 10 – 20 Hz (higher-frequency LFP in Figure 4) using a zero-phase fourth-order Butterworth filter with subsequent squaring of the filter output. The resulting estimate of the LFP power was smoothed with a normalized Gaussian window with a standard deviation of 2 s before computing the correlation with pupil dilation (Figure 4D). For visualization, LFP power was normalized such that low-frequency LFP power had a mean value of 1 (Figures 4A and 4B).

#### Construction of the camera system

We used a commercially available camera module (Adafruit 1937, Adafruit, USA) with an Omnivision OV5647 sensor capable of 640 × 480 pixels per frame at up to 90 Hz. The CMOS camera sensor has dimensions of 8.2 mm x 11.3 mm x 4.8 mm and weighs 0.5 g (including suspended part of the cable). The IR filter was removed to allow monitoring of behavioral variables in dark conditions using IR light. The sensor was attached to the neural implant using a custom camera holder. The camera holder consisted of a 3D printed frame with clips for holding the camera sensor (Figure S1A). A lightweight 21G steel cannula (thin wall cannula, length ∼ 2 cm, 0.04 g; Coopers Needle Works, UK) for holding the IR mirror (Calflex-X NIR-Blocking Filter, Optics Balzers, Germany; or 62-627 Hot Mirror, Edmund Optics, USA) was bent by about 75° in the middle, inserted with one end into a hole in the frame and fixed with epoxy resin (Araldite Steel, Araldite, UK). The mirror was cut to size 7 mm x 7 mm and attached to the cannula via a 3D printed holder. This enabled fine adjustment of the mirror relative to the camera sensor by moving the mirror along the cannula, rotating the mirror around the cannula, and also by further bending the cannula. A miniature connector (NSD-18-DD-GS, Omnetics, USA) for mounting the camera system to the implant was attached to the back of the 3D printed holder base using super glue (Loctite Power Flex Gel, Henkel, UK). After final adjustment of the mirror, either during surgery or during head-fixation of the animal on a running wheel (see “Neural recordings in head-fixed and freely moving mice”), the cannula and the mirror holder were permanently fixed using a thin layer of strong epoxy resin (Araldite Rapid, Araldite, UK). STL and OpenSCAD source files for the camera and mirror holders have been made freely available (see “Data and Software Availability”).

Illumination of the camera’s field of view, including eye and whisker pad, was provided by a small IR LED (VSMB2943GX01, Vishay, USA) mounted to either the bottom or the side of the camera holder, depending on the angle between camera sensor, mirror, and implant. The IR LED was powered by the headstage via two 36AWG wires and a small-package current-limiting resistor (Multicomp metric package size 3216, 100 – 180 Ohm, Farnell, UK). Custom cut gold pins (RS Pro Male (481-493) and Female (481-500) Solder D-sub Connector Contact, RS Components, UK) soldered to the wires and the headstage allowed quick and stable connection during experiments. All parts, including weight and estimated cost, are summarized in a separate step-by-step protocol (see “Data and Software Availability”). An example camera holder is shown in Figure S1C.

#### Interfacing with the camera

The camera was connected to a single-board computer (Raspberry Pi 3 model B, Raspberry Pi Foundation, UK) with ARM architecture and VideoCore 4 graphics processing unit (GPU). Data from the camera were read out with custom software using the Multi-media Abstraction Layer (MMAL) API (Broadcom Europe). Because miniature cameras such as the one used for the head-mounted system do not typically provide additional output signals to synchronize frame acquisition, we used the following approach to avoid dropped frames during recording and to obtain time stamps that were precisely synchronized with neural recordings. First, each frame was annotated with a time stamp from the GPU immediately after acquisition. Once the frame was received and decoded by the custom software, a TTL signal pulse was sent to the recording system using the general-purpose input/output capabilities of the single-board computer. The difference between the acquisition and TTL signal time stamps was saved to a separate file for post hoc alignment of TTL time stamps and neural data. Communication between the computer for recording neural data and the single-board computer for controlling the camera was done via ethernet using the ZeroMQ messaging library (http://zeromq.org/). Automatic starting/stopping of the camera system was controlled using a custom plugin for the open-ephys recording system (http://www.open-ephys.org/). Code for frame acquisition, TTL time stamp generation and alignment, and the plugin for controlling the camera have been made freely available (see “Data and Software Availability”).

Figures S1D and S1E demonstrates precision of aligned time stamps for a blinking LED (TSAL4400, Vishay, USA; typical rise/fall time 800 ns) recorded under the same conditions as the behavioral data in the experiments, for different video resolutions and frame rates. The LED was driven by a microcontroller (Teensy 3.2, PJRC, USA) and the same signal was sent to the recording system. The pixel corresponding to the maximum LED intensity was identified and LED onset times were extracted from the pixel intensity trace by thresholding at 0.5 full intensity.

#### Detection of camera image movements

For each recording, movement of the camera image was detected by selecting a region of interest (ROI) that contained a part of the neural implant (inset in Figure 2A). A correlation-based algorithm (Dubbs et al., 2016) was used to detect movements between the average ROI (averaged across all recorded images) and the ROI for each video image. Using the average ROI as reference image ensured that whisker or hair movements on single images did not have an impact on the overall detection performance. Images with changes in brightness exceeding three standard deviations were excluded from the analysis to remove periods when the camera view was blocked, e.g., during grooming. On average only 0.6% and 0.2% of the camera images were removed from the freely moving and head-restrained recordings based on this criterion, respectively.

#### Extraction of pupil positions from camera images

In order to perform tracking of pupil positions, it was necessary to remove bright regions from the camera image resulting from reflections of the illumination IR LED on the cornea. Therefore, contiguous bright regions on the recorded camera frames were detected by thresholding, and a binary mask was generated. Thresholds were manually selected for each session to include the major IR LED reflections. The original frame and the binary mask were used to estimate the values of masked pixels using non-texture image inpainting (März, 2011). An ellipse was fitted to the processed frame by thresholding, contour extraction, and least-squares ellipse fitting (Fitzgibbon et al., 1999). Contour extraction thresholds were manually adjusted for each session and only ellipses with mean pixel intensities below a user-defined threshold and with areas above another user-defined threshold were kept to reduce false positive rates. Thresholds were selected based on a small number of eye frames (≤2%) randomly selected from the whole recording. Finally, ellipses were manually verified using custom software including a graphical user interface. Ellipse-fitting code has been made freely available (see “Data and Software Availability”).

In experiments where we tracked the eye position in the dark, we administered an eye drop of physostigmine salicylate (0.1%–0.2%) 30 min in advance to limit pupil dilation (see for example Oommen and Stahl, 2008).

In experiments where we tracked both eyes simultaneously using a dual-camera head-mounted system, we analyzed pupil position and size independently for each eye. Video S1 shows an example of simultaneous recordings of both eyes in a freely moving animal. While the absolute size of the pupil differs between the two eyes in this example (presumably due to differences in shadowing of the two eyes by the multielectrode implant), pupil size fluctuations in the two eyes were strongly coupled (R = 0.81). Similar coupling was observed during head fixation in the same mouse and same recording session (R = 0.87).

Changes in pupil diameter in freely moving mice may be related to changes in behavioral state but also to changes in luminance due to self-generated movement. There are several possible ways to control for the effects of luminance changes in order to study the relationship between behavioral state and pupil diameter in freely moving animals. One approach is to minimize pupil fluctuations due to changes in luminance by restricting analysis to periods in which the animal is still. For example, in Figure 4D, we analyzed correlations between LFP power and pupil diameter for recording segments during which the mouse kept its head in a constant position for at least 15 s. A second approach is to perform (control) experiments in the dark to exclude any effects of luminance on pupil size, using pharmacology to enable pupil tracking (see above and Figure 5). A third, and more general, approach is to measure local luminance levels directly by adding to the head-mounted system an ambient light sensor (photodiode) that is sensitive to visible light (see Figure S4; TEMD5510FX01, Vishay USA; weight 0.04 g). The light sensor data can then be used to control for variations in pupil diameter due to luminance changes, for example by comparing trials with matched light levels or by performing partial correlations or using model-based approaches to correct for the estimated effects of luminance changes on pupil size.

#### Extraction of whisker pad movement from camera images

Movement of the whisker pad was extracted by selecting a rectangular region of the camera image containing the whisker pad. Dense optical flow was computed (Farnebäck, 2003) and the average optical flow across all pixels was used as a measure of whisker pad movement in horizontal (related to azimuth) and vertical (related to elevation) directions. All analyses in this study were based on horizontal movements.

We compared whisker pad movements recorded using the head-mounted camera (60 Hz) to data recorded simultaneously using an external camera (100 Hz) from above while the mouse was head-fixed. The head-mounted camera was able to capture important aspects of whisking including the whisking frequency and fluctuations in whisking envelope (Figure S5).

In some experiments described here (e.g., Figures 4 and S6), the camera system was operated with a frame rate of 30 Hz, and therefore whisker pad movements were measured only up to 15 Hz. In principle, however, the camera could be run at 90 Hz frame rates to capture more detailed aspects of whisking (e.g., whisker angles), using more sophisticated algorithms to extract these parameters at high frame rates (Perkon et al., 2011).

#### Extraction of head orientations from accelerometer signals

Gravity components in the accelerometer signals were estimated by low-pass filtering each channel with a zero-phase second-order Butterworth filter with a cut-off frequency of 2 Hz (Pasquet et al., 2016). Pitch, defined as the angle between the naso-occipital axis and the horizontal gravity plane, was extracted by computing the angle between the gravity vector and the y/z plane with normal vector $e_{x}={\left(1,0,0\right)}^{T}$. Roll, defined as the angle between the interaural axis and the horizontal gravity plane, was extracted by computing the angle between the gravity vector and the x/z plane with normal vector $e_{y}={\left(0,1,0\right)}^{T}$.

To compute head orientation maps (Figures 3F, 3I, and 4F) the low-pass filtered accelerometer signals were transformed into spherical coordinates (with elevation angle Θ and azimuthal angle Φ). A 2D histogram of head orientation vectors with a bin size of 5° for both elevation and azimuth was computed on the unfolded sphere. In order to visualize the histogram on a sphere, the number of samples within a each bin was normalized by the corresponding quadrangle area. Normalized histogram data were color-coded on a logarithmic scale.

#### Behavioral segmentation

Behavioral states were segmented using a semi-automatic classification algorithm. In a first step, about 1–2 hours of video recorded using external CMOS cameras (DMK 23UV024, The Imaging Source, Germany; 20–50 Hz frame rate) were annotated manually for each mouse and for each condition (“Implant+cam” and “Implant” in Figure 3). Only behavioral segments with a duration of at least 2 s were assigned a behavioral state.

The behaviors that we categorized were “grooming” (G), “eating” (E), “quiescence” (Q), and “active exploration” (A). Grooming comprised different stereotypical movements, e.g., movement of the forepaws over the nose and muzzle, strokes of forepaws across vibrissae and eye, and strokes with the hindleg. These movements were typically periodic and therefore easily distinguishable from the other behaviors. Eating was identified during chewing on seeds added to the environment. As chewing was also evident as artifacts on electrode channels, we used this information during manual annotation but not during automatic segmentation. Because the sessions in which seeds were added to the environment were not balanced across conditions, we accounted for this during the analysis shown in Figures 3E and 3H by assigning the mean value across sessions with seeds to those without seeds. Periods when the mouse was still for at least 2 s were classified as quiescence and periods when the mouse was exploring the environment and not grooming or eating were classified as active exploration.

We found that segmentation based on the time-domain accelerometer signals (Venkatraman et al., 2010, Dhawale et al., 2017) resulted in relatively low accuracy of identification of the behaviors described above. We therefore developed an algorithm performing segmentation in the frequency domain that considerably increased accuracy compared to segmentation based on time-domain signals (Figures S3D and S3E). The algorithm worked as follows: accelerometer signals (Figure 3B) were transformed into a spectro-temporal representation using a short-time Fourier transform (STFT) with a Hann window of length 2 s and a window shift of 40 ms. At each time step, the log-scaled magnitude of the transformed accelerometer signals was recast as a single vector containing data from all accelerometer channels. The middle point of the window was used as reference point for the annotated behavioral category. A Multilayer Perceptron (MLP) with one hidden layer (n = 100 hidden units with rectified-linear activation functions) was then fit to the data. The network was trained using the backpropagation algorithm and the weights were optimized using a stochastic gradient-based solver with adaptive momentum estimation (Kingma and Ba, 2014) via the sklearn Python package (Pedregosa et al., 2011).

We evaluated the prediction performance of the model using cross-validation. That is, the dataset was divided into 4 parts, model parameters were estimated leaving out one of the parts, and the predictive quality of the model fit was evaluated on the part left out. This procedure was repeated leaving out each of the 4 parts in turn and the prediction accuracy averaged to yield an estimate of the goodness-of-fit of the model. The confusion matrices in Figures 3D and S3A show the cross-validated true positive rate computed from the manually annotated data (“Human observer”) and the prediction of the model.

To assess the differences between occupancies of the different states in the two experimental conditions (“Implant+cam” and “Implant” in Figures 3E and 3H) we computed the least absolute deviation (L1 norm) between the distributions for both conditions. To confirm the significance of this difference, we used a permutation test. A null distribution was generated by shuffling “Implant+cam” and “Implant” condition labels across recording sessions. This approach ensured that any significant differences from the null distribution could be attributed to the presence of the camera rather than time of the recording session (see Figures S3B and S3C). The permutation procedure was repeated 10000 times, and a *P*-value was generated by computing the fraction of permutations with least absolute deviations larger than the value computed on the original dataset. The same permutation procedure was used to determine the significance of the difference between body speed distributions in the active state (Figures 3G and 3J). Mean and variance of head orientations (Figures 3F and 3I) were computed using a permutation test for the difference in circular mean and variance as test statistic, respectively.

#### Sound presentation

Broadband noise burst stimuli (50 ms, 50 or 55 dB SPL, noise bust rate 0.5 Hz or 1 Hz) were generated using custom software, converted to an analog signal (HDSPe AIO, RME, Germany), amplified (RB-850, Rotel, Japan), and delivered via a loudspeaker (XT25TG30-04, Tymphany, USA) mounted about 1 m above the circular environment. Sound pressure levels of the acoustic stimuli were measured (40BF 1/4 inch free-field microphone and 26AC preamplifier, GRAS, Denmark) and calibrated to the center of the circular environment. In the experiments shown in Figure S6, recordings with and without acoustical stimulation were interleaved (up to 5 min each, total duration 30 min) during periods when the animal was quiescent and immobile.

#### Prediction of eye position using head orientation

Pupil positions were extracted from video data (sampled at 42–60 Hz) as described in “Extraction of pupil positions from camera images.” Only time points at which the pupil could be detected were included in the analysis and no smoothing was applied for the analysis. For visualization, extracted eye position and pupil dilation traces were smoothed using a 3-point Gaussian window with coefficients (0.072, 0.855, 0.072). Head pitch and roll were computed from signals recorded using the 3-axis accelerometer (sampled at 7500 Hz) integrated into the neural recording as described in “Extraction of head orientations from accelerometer signals.”

For each pupil position $p_{i},\;i=1,2,\ldots ,N$, the most recent history of each signal within a time window of 500 ms was recast as vector $u_{i}$, $v_{i}$ for pitch and roll, respectively. Linear interpolation was used to find the pitch/roll at time lags −500,−475,−450,…,0 ms.

Two different models were trained using the resulting data. The linear model assumes that pupil positions are related to the pitch and roll via

(Equation 1) $${\hat{p}}_{i}=k_{\text{pitch}}^{T}u_{i}+k_{\text{roll}}^{T}v_{i}+k_{0}+N\left(0,\sigma^{2}\right).$$

The linear weighting vectors $k_{\text{pitch}}$ and $k_{\text{roll}}$, and the offset term $k_{0}$ were found using a Bayesian method for determining the relevance of inputs, known as Automatic Relevance Determination (ARD) (MacKay, 1996). Because the relation between accelerometer signals and pupil position can potentially be nonlinear we also tested a Multi-Layer Perceptron as described in Results.

In some experiments we added a lightweight gyroscope sensor (MPU-9250, InvenSense, USA, weight 0.25 g) to measure angular velocity, including rotations about the yaw and pitch axes (see Figures 6A and 6B). The sensor was calibrated using a stepper motor (Adafruit 324, Adafruit, USA) and a contact tachometer (DT-2235B, Lutron Electronic, Taiwan). To approximate angular yaw position we convolved the velocity signal with an exponential decay function with time constant τ=1 s and extended the models to also include the recent history of angular positions (Equation 1; Figure S7E).

The prediction performance of the different models was evaluated using cross-validation as described above (but with n=5 fold). Similarity between predicted and measured eye positions was quantified using the coefficient of determination $R^{2}=1-\text{rss}/\text{tss}$ where rss is the residual sum of squares and tss is the total sum of squares.

In behavioral experiments where we tracked the eye position in the dark (see “Extraction of pupil positions from camera images”), we typically recorded 2–3 segments (10 min each) before administration of an eye drop of physostigmine salicylate in a lit environment (“Light” in Figure 5F) and one recording (10 min) in the dark about 30 min after administration of the eye drop (“Dark” in Figure 5F). This strategy was adopted because after about 20 min in the dark, the pupil size became too large to allow for reliable tracking without pharmacological constriction. This procedure was repeated on four different days in one mouse resulting in 10 recordings with light on and 4 recordings in the dark.

#### Analysis of head movement onsets

Data for analysis of head movement onsets was collected while mice were exploring a circular environment (see “Neural recordings in head-fixed and freely moving mice”). The bottom of the circular environment consisted of an acrylic sheet that allowed reliable tracking of the mouse’s body using a camera placed below the environment, even in the presence of headstage and camera cables. Head movements were extracted from accelerometer signals by subtracting the gravity components (see “Extraction of head orientations from accelerometer signals”). The magnitude of head movements was computed as

(Equation 2) $$\left|a\left(t\right)\right|=\sqrt{a_{x}{\left(t\right)}^{2}+a_{y}{\left(t\right)}^{2}+a_{z}{\left(t\right)}^{2}},$$

where $a_{x}$, $a_{y}$, and $a_{z}$ are the head acceleration components along x, y, and z channels of the accelerometer, respectively, sampled at time step *t*. The magnitude was smoothed using a low-pass filter with a cut-off frequency of 2 Hz and thresholded using a fixed threshold across all mice and recordings (0.0625 g). Positive threshold crossings were classified as head movement onset if the smoothed magnitude of the accelerometer signals was (i) below the threshold for at least 0.5 s before and (ii) above the threshold for at least 0.5 s after the threshold crossing (Figure 7B). Moreover, movement onsets during locomotion periods (body speed ≥ 1 cm/s) were excluded from the analysis in Figure 7. Onsets of whisker pad movements and locomotion were computed in the same way as head movement onsets but whisking thresholds were selected separately for each mouse and the minimum duration above threshold was 0.1 s to account for faster movements of whiskers. Because freely moving mice ran only occasionally during each recording session, presumably due to the relatively small size of the circular environment, we computed locomotion onsets for head-fixed mice running on a cylindrical treadmill (threshold 1 cm/s) in the dark.

For the analysis, spike times were aligned to head movement onsets for each recorded V1 cell. To quantify the extent to which head-movement-related activity modulated the activity of each cell, we computed a modulation index (MI) defined as

(Equation 3) $$\text{MI}=\frac{N_{\text{post}}-N_{\text{pre}}}{N_{\text{post}}+N_{\text{pre}}},$$

with $N_{\text{pre}}$ and $N_{\text{post}}$ denoting the average number of spikes 1 s before and 1 s after movement onset, respectively. MI values reported here were computed without subtraction of the baseline firing rate.

Because tracking of the pupil in the dark can be challenging due to increased pupil dilation (and because the effect of pharmacological intervention to reduce pupil dilation is not known, see “Extraction of pupil positions from camera images”), we extracted initial eye movements after movement onsets by measuring optical flow of the pupil edges in the dark. The region of the camera image containing the eye was filtered using a median filter with a window length of 15 pixels before computing optical flow of the pupil edges. This step ensured that movements of hair or IR LED reflections did not impair optical flow measurements. The flow for each pixel was computed using the same dense algorithm as for the whisker pad movements. To convert optical flow (measured in pixels per frame) to horizontal and vertical eye positions, we integrated the average flow for each dimension across time (i.e., frames). The integrated flow provides an approximation to initial eye movements after a head movement onset (but might diverge after some time due to potentially leaky integration of the flow measure). Comparing flow-based pupil positions to direct pupil fitting in dim light conditions (i.e., when the enlarged pupil was still possible to identify using ellipse-based pupil fitting), we found that analysis of optical flow of pupil edges yielded reliable estimates of eye positions after head movement onsets in the dark (Figures S8G–S8I).

To test whether different types of eye movements had an effect on the observed head movement-related modulation of V1 firing, we divided head movement onsets into two groups: those accompanied by eye movements in the direction predicted from the head movement based on the models described above, and those accompanied by eye movements that were not consistent with model predictions. Because the observed modulations of V1 firing were fast (typically appearing less than 100 ms after the head movement onset), we used the x/y values of the earliest peak/trough in the eye movement trace within 100 ms after head movement onset as an approximation to the initial eye movement. This yielded one vector (i.e., x/y pair) for the measured and one vector for the predicted eye movement trace following a head movement onset. Only pairs with maximum/minimum within 100 ms after the head movement onset were included in the analysis. The values in Figure 7E show the cosine similarity (i.e., the cosine of the angle) between the measured and predicted eye movement vectors.

### Quantification and Statistical Analysis

Specifics on the statistical methodologies and software used for various analyses are described in the corresponding sections in Results, figure legends, STAR Methods, and supplemental figures. Statistical test results are described as significant in the text where p < 0.05.

### Data and Software Availability

Software to control the camera and to perform data extraction, along with 3D models for custom parts in the camera system and a step-by-step construction protocol, have been made available at http://www.gatsby.ucl.ac.uk/resources/mousecam/. Further data from this study are available from the corresponding authors upon reasonable request.

## Supporting Citations

The following reference appears in the Supplemental Information: Hussain et al. (2009).

## Supplemental Information

Document S1. Figures S1–S8

Document S2. Article plus Supplemental Information
