# Supplementary material for: A Head-Mounted Camera System Integrates Detailed Behavioral Monitoring with Multichannel Electrophysiology in Freely Moving Mice
Source: Neuron. 2018 Oct 10;100(1):46–60.e7. doi: 10.1016/j.neuron.2018.09.020 (PMC6195680; doi:10.1016/j.neuron.2018.09.020)
Supplement: Document S1. Figures S1–S8 [file mmc1.pdf]

Neuron, Volume *100*

Supplemental Information

A Head-Mounted Camera System Integrates Detailed Behavioral Monitoring with Multichannel Electrophysiology in Freely Moving Mice

Arne F. Meyer, Jasper Poort, John O'Keefe, Maneesh Sahani, and Jennifer F. Linden

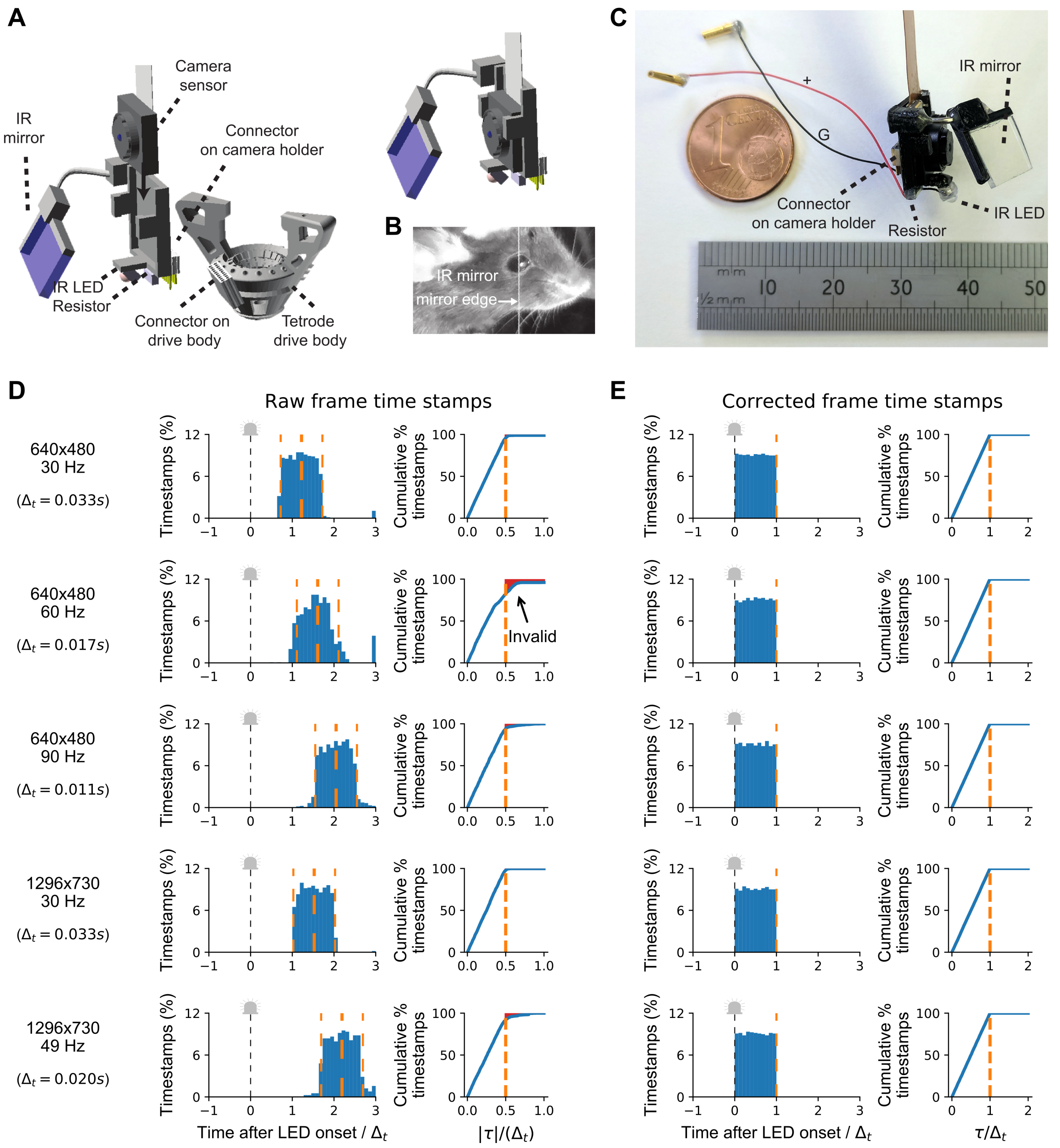

Figure S1: Design of the miniature head-mounted camera system and frame synchronization. Related to Figure 1. (A) 3D computer-aided design (CAD) model showing the entire camera assembly, including camera sensor, camera holder, IR mirror, IR LED, miniature connector on the camera holder, and complementary connector permanently fixed to the drive body of the tetrode implant. The camera sensor is mounted on the camera holder via small clips on the sides of the 3D-printed holder. Connectors allow the camera system to be attached to the neural implant for recording sessions, and removed otherwise. (B) IR mirror is largely transparent to visible light. Example image with 25 x 25mm Calflex-X (>80% transmission) IR mirror (edge indicated) with visible light illumination. (C) Example camera holder. Black and red wires are connected to ground (G) and positive (+) pins on the headstage (respectively), to provide power to the IR LED via the current-limiting resistor. Euro cent coin (left) and ruler (bottom) are shown for size comparison. (D) Distributions of raw frame timestamps (left) generated using the single-board computer controlling the head-mounted camera. Timestamps were normalized by the frame interval of the camera (Δ_t) and mean and $\pm\frac{1}{2}\Delta_t$ are indicated by the solid and dashed orange lines, respectively. The cumulative fraction of timestamps with time $\pm\tau$ around the mean (right) reveals that not all timestamps were generated within the interval $\pm\frac{1}{2}\Delta_t$ as indicated by the shaded red area. Therefore using the mean delay does not allow for reliable and precise synchronization of camera and neural data. Each row represents results for the video format shown on the left. (E) The same but after correcting video timestamps as described in STAR Methods. All frame timestamps were within the frame interval $[0, \Delta_t)$.

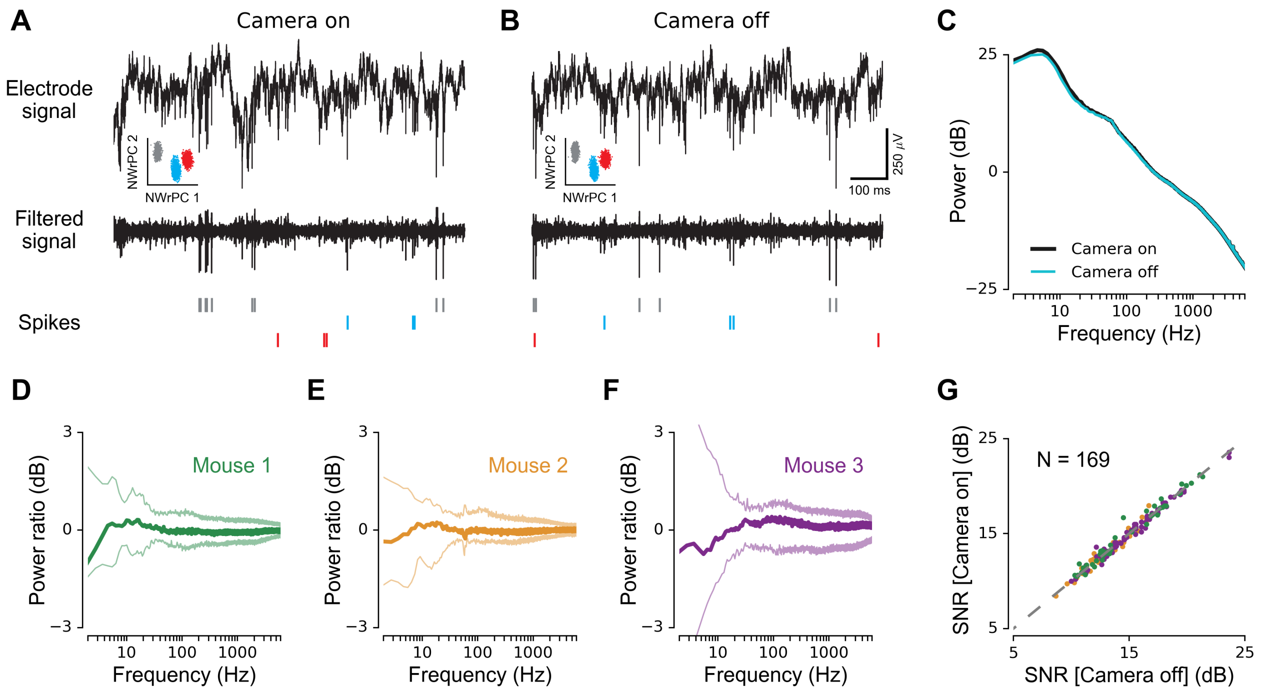

Figure S2: Neural recording quality with head-mounted camera. Related to Figure 1 and STAR Methods. (A) Broadband electrode signal from an example tetrode channel (top; hardware filtering 1.1 — 7603.8 Hz), high-pass filtered signal (middle; high-pass cutoff 600 Hz), and extracted spikes (bottom) with head-mounted camera activated (powered and operating "camera-on" condition). Inset shows projections of spike waveforms for identified cells into the space defined by the first two noise-whitened robust principal components (NWrPC 1 and 2). (B) The same as in A but with head-mounted camera switched off ("camera-off" condition). Scales apply to bottom and top traces in A and B. The isolation of action-potential spikes appeared unchanged with the camera on or off, and the projection of spike waveforms into a noise-whitened robust PCA (NWrPC) space (Sahani, 1999) was similar in both conditions (insets in A,B). (C) Power density spectrum of broadband electrode signals for camera-on and -off conditions (10 minutes each) recorded in the same session. Note that the two lines are closely overlapping. (D-F) Mean log power ratio between broadband electrode signals in camera-on and -off conditions (counter-balanced, 10 minutes each condition per session) across recording sessions for three mice (mouse 1 & 2, $n = 9$; mouse 3, $n = 19$). Pale lines indicate standard deviation of log power ratios for recordings in camera-off condition alone. Across the frequency range of neural signals (2 to 6000 Hz), differences in log-power ratios were close to 0 dB, and within one standard deviation of the within-condition (camera-off) variability. (G) The signal-to-noise ratio (SNR; see STAR Methods) between identified spike waveforms and noise in the high-pass filtered signal, was not different for camera-on versus camera-off conditions (Wilcoxon signed-rank test, P=0.19, 158 single-units, 11 multi-units).

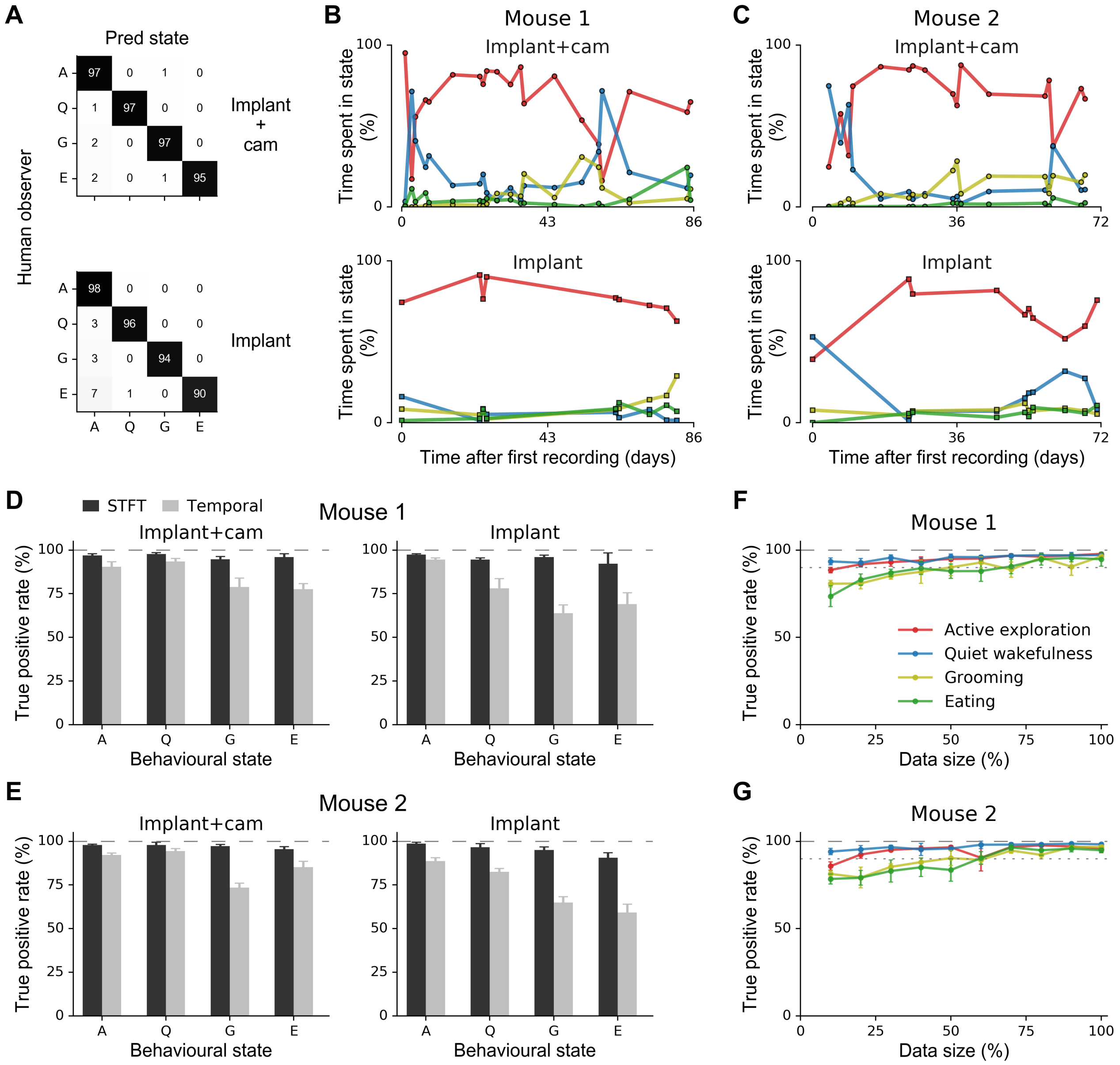

Figure S3: Details on behavioral segmentation. Related to Figure 3. (A) Confusion matrix showing cross-validated classification performance for second mouse. Top: Mouse with implant and camera. Bottom: Mouse with implant only. (B,C) Time spent in the different behavioral states as a function of days after first recording for the two mice. Top: Mouse with implant and camera. Bottom: Mouse with implant only. Same legend as in F. (D,E) Cross-validated segmentation accuracy (true positive rate) for the temporal accelerometer representation ("Temporal", gray bars) used in Venkatraman et al. (2010) and the spectral (short-term Fourier transform) representation proposed in this study ("STFT", black bars) for the two mice, both with and without camera. Total time of annotated data: "implant+camera", 90 minutes for mouse 1 and 60 minutes for mouse 2; "implant": 60 minutes for mouse 1 and 60 minutes for mouse 2. (F,G) Cross-validated segmentation accuracy as a function of training data size for the "implant + camera" condition. 100% corresponds to 58 minutes and 45 minutes of recorded data for mouse 1 and 2, respectively. Validation set sizes were 19 and 15 minutes for mouse 1 and 2, respectively, and validation was performed using the same 4-fold cross-validation scheme as in D and E. The results suggest that about 75% of the annotated training data are sufficient for behavioral segmentation with >90% accuracy (gray dotted line).

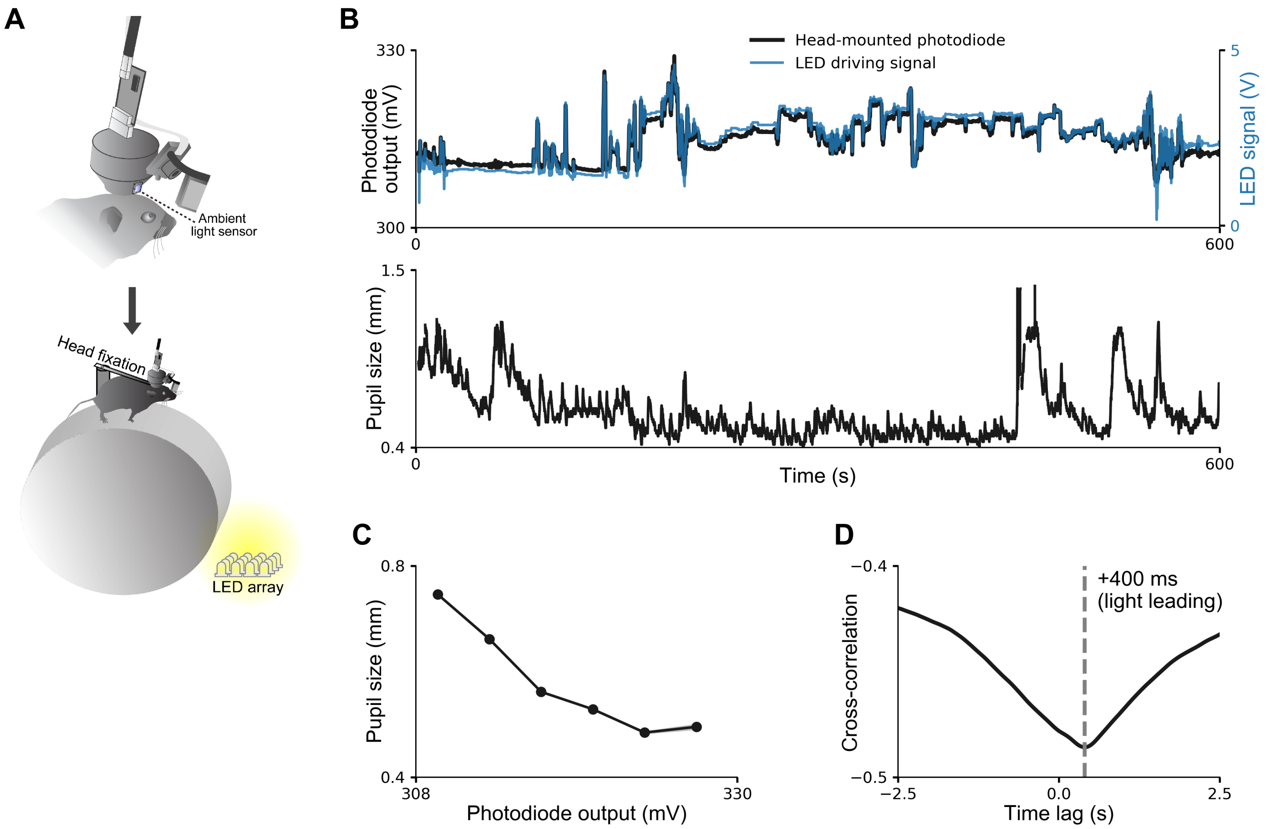

Figure S4: Measurement of relationship between ambient light level and pupil size. Related to Figure 4 and STAR Methods. (A) Top: an ambient light sensor (photodiode) was attached to the implant above the eye to record variations in luminance while pupil fluctuations were monitored using a head-mounted camera. Bottom: ambient light levels were varied for head-fixed mouse using a LED array (visible light). (B) Top: LED driving signal (blue) and measured photodiode signal (black). Photodiode output shows close correspondence between measured light level and signal driving LEDs (cc=0.98). Bottom: Pupil size. (C) Relationship between photodiode output and pupil size for the data in B. Plot shows mean ±SEM (smaller than line width). (D) Cross-correlation between photodiode output and pupil size for the data in B. Changes in light signal led pupil fluctuations by 400 ms, in agreement with previous work on pupillary response latency in mice (Hussain et al., 2009).

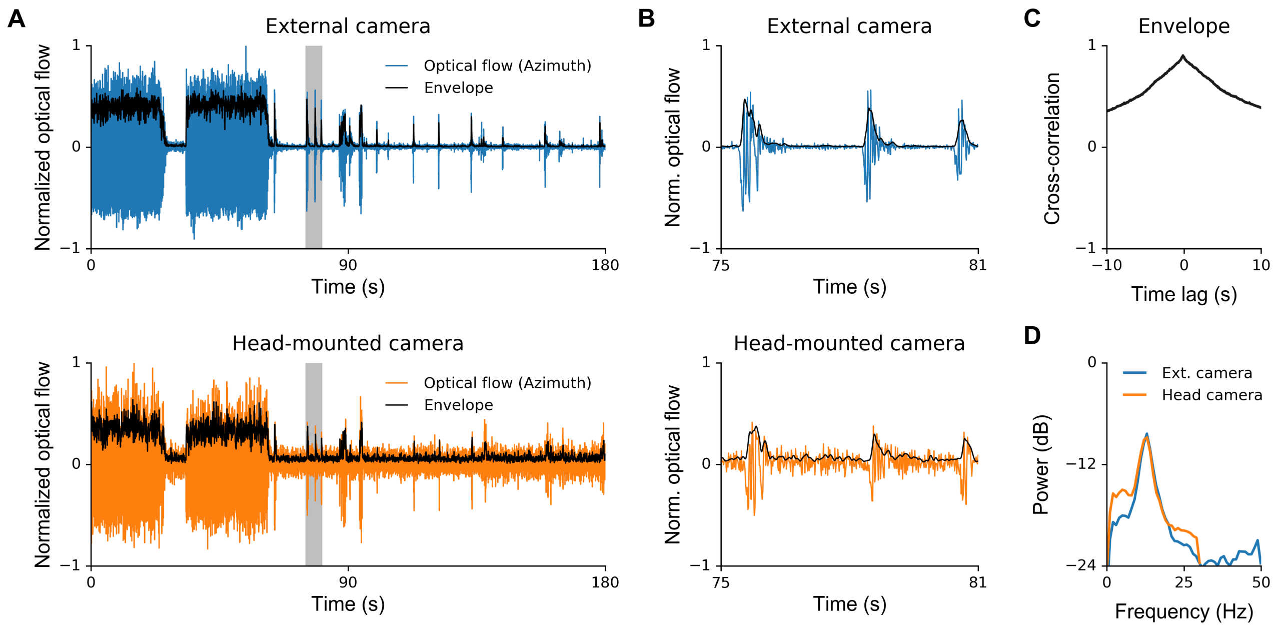

Figure S5: Correspondence between whisker pad movement estimates obtained from head-mounted and external camera images. Related to Figure 4. (A) Top: Optical flow extracted from external camera images of the whiskers viewed from above in a head-fixed mouse; sampling rate 100 Hz. Bottom: Optical flow extracted simultaneously from head-mounted camera images of the whisker pad; sampling rate 60 Hz. Optical flow was normalized to the interval $[-1, 1]$. Black lines indicate envelope obtained by low-pass filtering the magnitude of the optical flow with a causal filter with a cutoff frequency of 5 Hz. (B) Zoomed-in traces for gray shaded region in A. (C) Cross-correlation function between envelopes extracted from the external camera signal and the head-mounted camera signal in A. (D) Power spectral densities estimated from the optical flow signals in A.

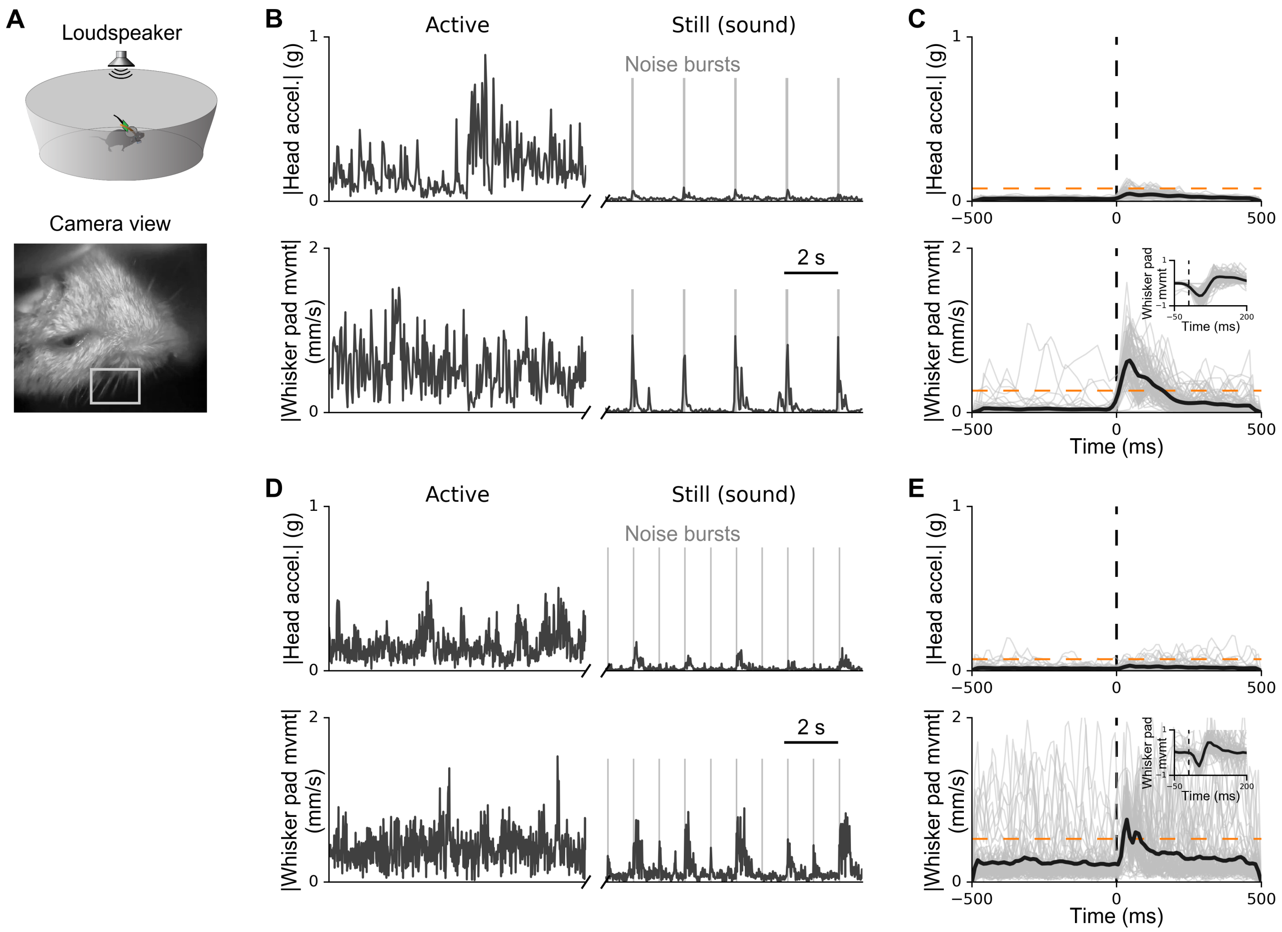

Figure S6: Sound-evoked whisker movements measured with the head-mounted camera. Related to Figure 4. (A) Top: Sounds were presented via a loudspeaker mounted 1 meter above the center of the circular environment. Bottom: Example frame from head-mounted camera focused on whiskers from above (gray rectangle). (B) Example traces showing head movement (top) and whisker pad movement magnitude (bottom) for an active period when the mouse was exploring the environment, and a still period when the mouse was immobile. During the still period, noise bursts (55 dB SPL, 50 ms) were presented every 2 seconds. (C) Sound onset-triggered head movements (top) and whisker pad movements (bottom). The dashed orange line indicates one standard deviation for movements observed in the active period, for comparison. Sound-evoked whisker movements follow a stereotypical protraction/retraction movement pattern (inset). (D,E) The same as in B,C but for second mouse. Noise bursts (50 db SPL, 50 ms) were presented every second.

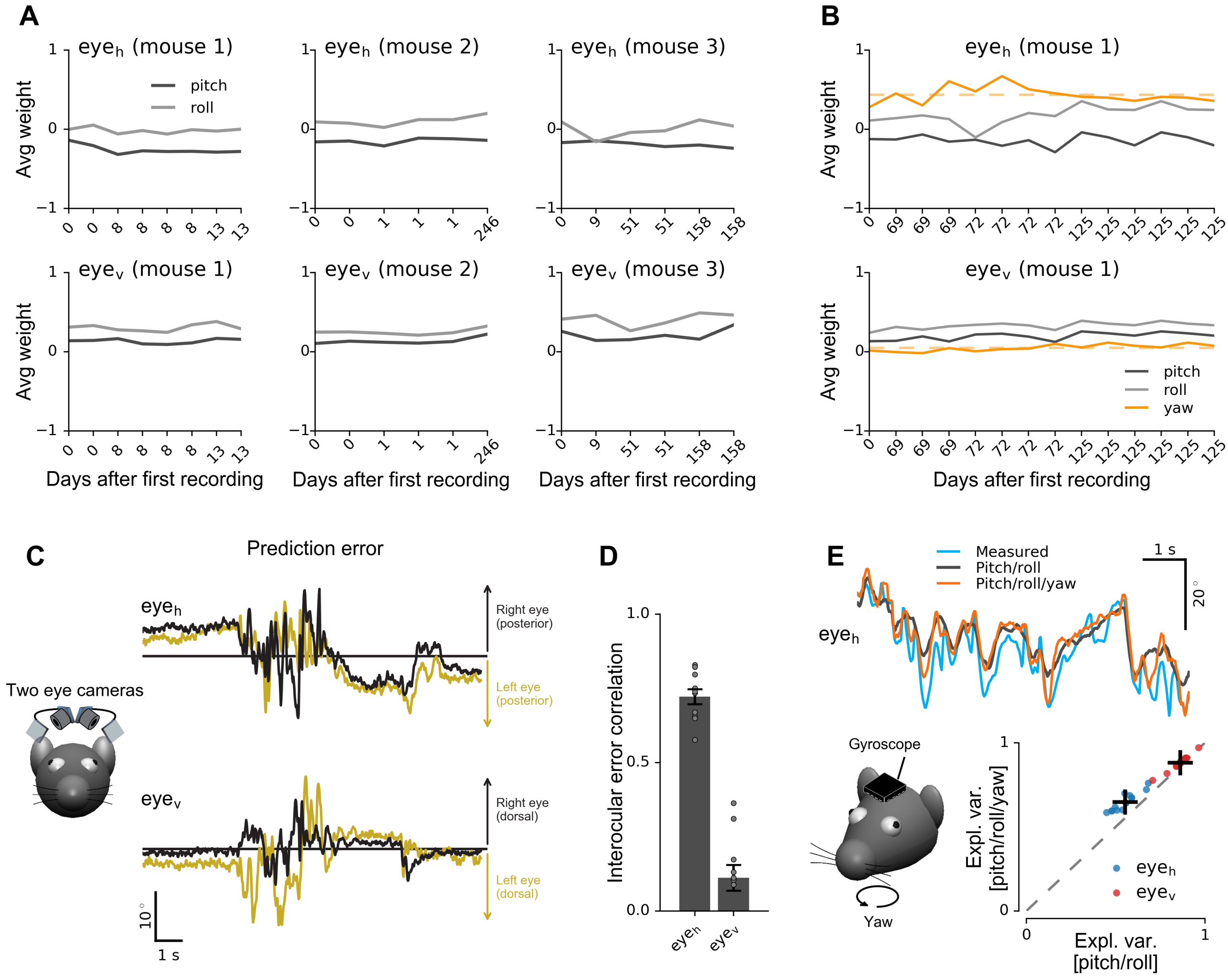

Figure S7: Details on prediction of eye position from head orientation. Related to Figure 5. (A) Average weights of linear model as a function of time after first recording, illustrating stability of estimated weights across recording sessions. Top and bottom rows show weights for horizontal and vertical eye position, respectively. Repeated recording days indicate multiple recordings (10 minutes each) on the same day. Same data as for the three mice shown in Figure 5E. (B) The same as in A but for 14 recordings in one mouse with accelerometer and gyroscope sensors (yaw). (C) Simultaneous monitoring of both eyes using two head-mounted camera systems in a freely moving mouse. Example traces show prediction errors of the pitch/roll-based nonlinear model for horizontal (top) and vertical (bottom) eye position. Yellow lines, left eye. Black lines, right eye. (D) Correlation between model prediction errors for the two eyes, for 6 experiments in one mouse. Results indicate that failures to predict horizontal eye position based on head orientation (pitch/roll) were strongly correlated between the two eyes. (E) In addition to head acceleration, rotations about the yaw axis were measured using a head-mounted gyroscope (bottom left). Example traces show predictions of a nonlinear model trained with pitch/roll (black) and pitch/roll and yaw rate (orange). Cross-validated prediction performance increased mostly for horizontal eye position (bottom right). Increases in explained variance were statistically significant for both horizontal and vertical eye position, and for both linear and nonlinear models (Wilcoxon signed-rank test, $P < 1 \cdot 10^{-3}$; $n = 14$ recordings; 10 minutes each).

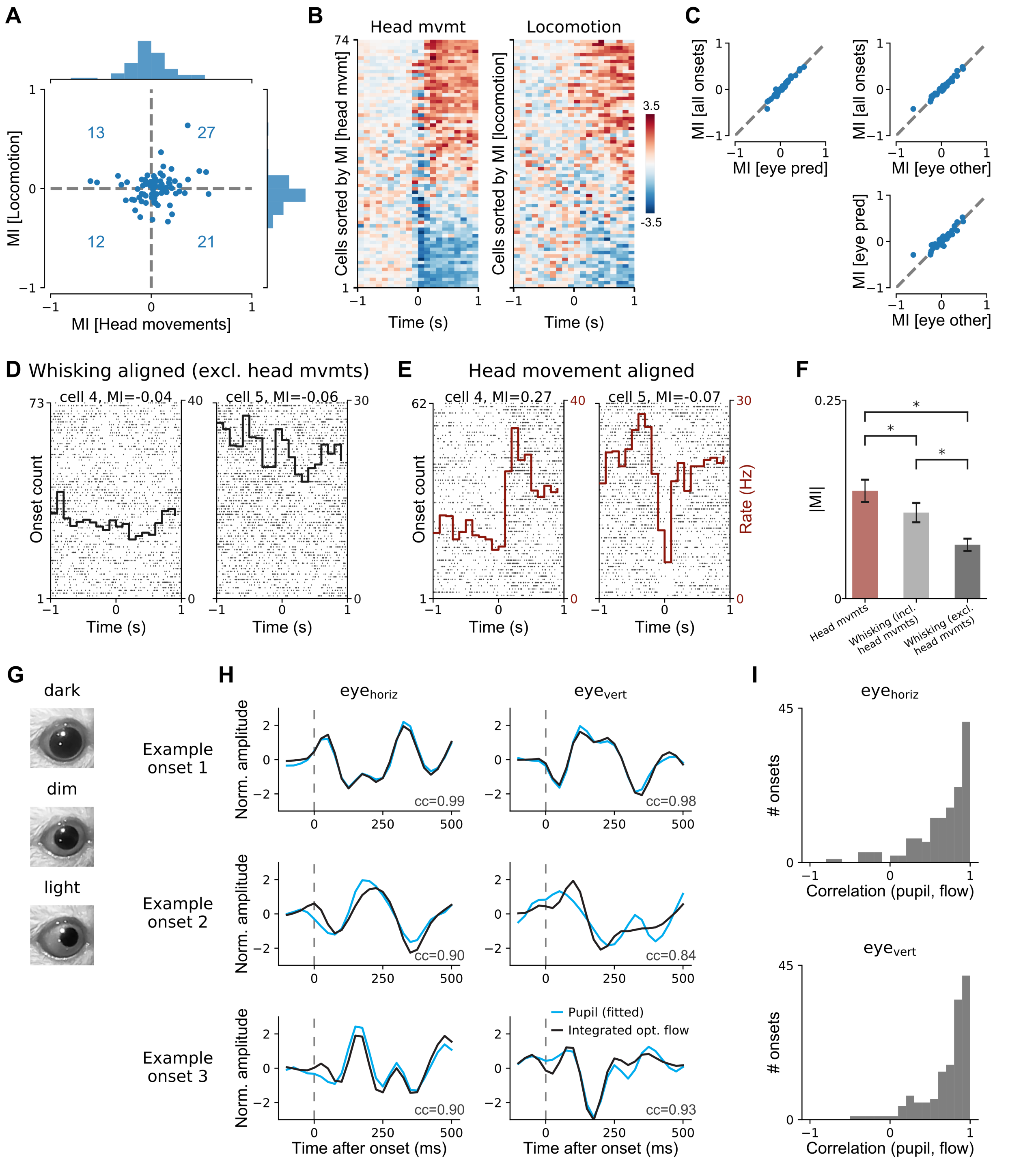

Figure S8: Modulation of V1 activity by head movements versus locomotion, whisking, or eye movements. Related to Figure 7. (A) Relationship between modulation indices (MIs) for V1 spike trains either recorded when mice were head-fixed on a cylindrical treadmill and aligned to locomotion onsets, or recorded when mice were unrestrained but immobile (body speed < 1 cm/s) and aligned to head movement onsets. Plot shows data from all V1 cells recorded in 3 mice which had firing rates of at least 2 spikes/s in both conditions. Histograms show marginal MI distributions for head movement onsets (top) and locomotion (right). Blue numbers indicate absolute numbers of cells in each quadrant. (B) Peristimulus time histograms (PSTHs) for spike trains aligned to head movement onsets (left) and locomotion onsets (right) for the same cells as in A. For visualization, each PSTH was transformed to a z-score by subtracting from it the mean of the pre-onset activity and dividing by the standard deviation for the entire PSTH. Cells were sorted by MIs computed for each condition. (C) Comparison of the MI values for the same V1 spike trains for head and eye movements in which the eye movement was predictable ("Eye pred") or *not* predictable ("Eye other") from the head movement. (D) Spike rasters and rate histograms for recordings from two V1 cells, aligned to whisker

Figure S8 (Cont.): pad movement onset after excluding periods of head movements. Other conventions as in Figure 7C. (E) The same cells as in H, but with spike times aligned to head movement onsets. (F) Comparison of absolute MIs for V1 activity when aligned to head movement onsets (red bar), whisking onsets when head movements are included (light gray bar), and whisking onsets excluding periods of head movement (dark gray bar). Plot shows mean $\pm$ SEM across 16 recordings (10–40 minutes each) in 3 mice. (G) Optical flow-based pupil movement extraction. Frames showing typical pupil dilation in dark (top), dim light (middle), and normal light (bottom) conditions. Dim and normal light conditions allowed direct fitting of pupil position. (H) Example horizontal and vertical eye movement traces extracted in the dim light condition, either by fitting an ellipse to the pupil (blue line) or by integrating optical flow (black line) of the pupil edge. Trends were removed by high-pass filtering. (I) Correlations between movement onset-triggered ellipse-fitted and optical flow-based pupil position traces for ten minutes of recorded data (144 onsets).
